# The Effects of Separate Facial Areas on Emotion Recognition in Different Adult Age Groups: A Laboratory and a Naturalistic Study

**DOI:** 10.3389/fpsyg.2022.859464

**Published:** 2022-06-30

**Authors:** Larissa L. Faustmann, Lara Eckhardt, Pauline S. Hamann, Mareike Altgassen

**Affiliations:** Department of Psychology, Johannes Gutenberg University Mainz, Mainz, Germany

**Keywords:** emotion recognition, aging, holistic facial processing, middle-aged adults, older adults, face masks

## Abstract

The identification of facial expressions is critical for social interaction. The ability to recognize facial emotional expressions declines with age. These age effects have been associated with differential age-related looking patterns. The present research project set out to systematically test the role of specific facial areas for emotion recognition across the adult lifespan. Study 1 investigated the impact of displaying only separate facial areas versus the full face on emotion recognition in 62 younger (20–24 years) and 65 middle-aged adults (40–65 years). Study 2 examined if wearing face masks differentially compromises younger (18–33 years, *N* = 71) versus middle-aged to older adults’ (51–83 years, *N* = 73) ability to identify different emotional expressions. Results of Study 1 suggested no general decrease in emotion recognition across the lifespan; instead, age-related performance seems to depend on the specific emotion and presented face area. Similarly, Study 2 observed only deficits in the identification of angry, fearful, and neutral expressions in older adults, but no age-related differences with regards to happy, sad, and disgusted expressions. Overall, face masks reduced participants’ emotion recognition; however, there were no differential age effects. Results are discussed in light of current models of age-related changes in emotion recognition.

## General Introduction

The accurate perception of emotions is essential for human communication and adequate social functioning (McArthur and Baron, 1983; Avery et al., 2016). Individuals that are not able to identify and differentiate emotions in others may have difficulties reacting appropriately and communicating effectively. Deficits in emotion recognition are evident in different clinical populations, for example, traumatic brain injury (cf. a meta-analysis; Murphy et al., 2021) or neuro-psychiatric disorders such as schizophrenia (Mandal et al., 1998; Kohler et al., 2000). However, importantly, research suggests that with increasing age, even healthy adults may develop difficulties to correctly identify emotional expressions (Ruffman et al., 2008). Given the high relevance of successful social interaction and perceived involvement in a community for health and well-being across the entire lifespan (Cornwell and Waite, 2009), a clear understanding of possible changes in emotion recognition across adulthood and their underlying factors is needed.

### Processing of Faces in Older Adults

Empirical evidence suggests that faces are processed more holistically than other objects. For example, various facial features are automatically combined to form a unified whole (Farah et al., 1998; Calder et al., 2000; Maurer et al., 2002; Calder and Jensen, 2005). A common index of holistic processing is the “composite effect” (Young et al., 1987) which shows that upper and lower face halves interact perceptually and cannot be recognized independently. Even though, there is evidence that the basic ability to identify faces decreases with age (Searcy et al., 1999), no decline in the composite effect was found, suggesting that older adults use holistic identification strategies to the same extent as younger adults (Konar et al., 2013; Meinhardt-Injac et al., 2017; Boutet and Meinhardt-Injac, 2019). Possibly, the decline in identification accuracy is due to lower sensitivity to information conveyed by horizontal facial contours (Obermeyer et al., 2012; Pachai et al., 2013) or lower sensitivity to the horizontal spacing between features such as the eyes (Murray et al., 2010; Chaby et al., 2011). Importantly with regards to emotion recognition, Beaudry et al. (2014) found that both, holistic (the recognition of structural relations between different facial components) and featural processing (single facial features may suffice to recognize an emotional expression) may be involved—depending on the expressions being observed. The mouth area seemed to be important for the identification of happiness and the eye/brow area for the recognition of sadness, while results for all other basic emotions were less consistent. Fear was the only emotion that depended on holistic processing to be recognized (Beaudry et al., 2014).

### Emotion Recognition Across the Lifespan

Research indicates a decline in emotion recognition across the lifespan: A meta-analysis by Ruffman et al. (2008) showed reduced emotion recognition in at least some of the basic emotions across all tested modalities (faces, voices, bodies/contexts, and matching of faces to voice). The recognition of anger, fear, and sadness seems to be most affected by old age, while the recognition of surprise and happiness were less reduced; disgust was even better recognized by older adults. Similarly, a more recent meta-analysis by Hayes et al. (2020) showed strong age-related deficits in the recognition of anger, sadness, and fear, but less severe age-related difficulties in the identification of surprise and happiness, and spared recognition of disgust. However, these strong negative age effects in anger, sadness, and fear were only found when static stimuli were used. For dynamic stimuli (i.e., videos), older adults demonstrated only moderate age-related differences across all emotions. To date, it is still unclear at which point in the adult lifespan changes in emotion recognition performance occur. Isaacowitz et al. (2007) reported better emotion recognition in younger (18–39 years) as compared to middle-aged adults (40–59 years), but fewer or no differences between middle-aged and older adults (60–85 years). These findings suggest that decreases in emotion recognition may begin in middle adulthood. Mill et al. (2009) even found evidence for earlier negative age effects. In their study, already 21- to 30-year-old participants performed worse in the identification of sadness and anger than younger participants (18–20 years); though the decline was much stronger in 31- to 40-year-olds. Some studies (Riediger et al., 2011; Scherf and Scott, 2012) indicated that participants perform better in emotion recognition tasks when pictures of faces from their own-age group are being presented (i.e., an own-age bias, see Rhodes and Anastasi, 2012). For example, younger adults (20–31 years) performed worse than middle-aged (44–55 years) and older participants (70–81 years) when decoding emotional expressions by middle-aged or older adults as compared to adults of their own age (Riediger et al., 2011). This own-age bias has been attributed to participants’ typically more frequent exposure to individuals from similar age groups which may lead to greater expertise in identifying own-age faces. However, there are also findings that speak against the presence of an own-age bias and a general superior identification of emotional expressions from younger faces. For instance, Ebner and Johnson (2009) found younger (18–22 years) and older adults (65–84 years) to be better at identifying facial expressions of younger as compared to older faces (cf. similar findings, Malatesta, 1987; Borod et al., 2004).

### Causes of Age-Related Emotion Recognition Decline

Over the years, several explanations have been put forward for the altered emotion recognition in older adults. One prominent approach states that deficits in the identification of negative emotions (Wong et al., 2005; Keightley et al., 2006) and the spared recognition of positive emotions (Orgeta and Phillips, 2008; Murphy et al., 2010; Murphy and Isaacowitz, 2010) might be explained by the so-called “positivity effect.” The positivity effect describes a processing bias toward positive as compared to negative information in older adults, whereas younger adults demonstrate the opposite pattern (Kennedy et al., 2004; Carstensen, 2006). This bias has also been observed with regards to memory; older adults tend to remember more positive than negative information, whereas, in contrast, younger adults remember more negative than positive information (e.g., Ochsner, 2000). However, a meta-analysis by Ruffman et al. (2008) indicated that older adults neither always show difficulties with the recognition of negative emotions (e.g., no age differences in the recognition of vocal expressions of fear or disgust; better recognition of facial disgust) nor always exceed in the recognition of positive emotions (e.g., significantly worse recognition of facial and vocal expressions of happiness: Brosgole and Weisman, 1995; Sullivan and Ruffman, 2004; Wong et al., 2005; Keightley et al., 2006; Isaacowitz et al., 2007; Henry et al., 2008; cf. a recent review Ruffman, 2020).

Eye-tracking studies suggest that age-related differences in emotion recognition may be due to age-related differences in face exploration. Older adults tend to mainly look at the mouth region, whereas younger adults focus on the eye region (e.g., Wong et al., 2005; Sullivan et al., 2007). Importantly, the relative importance of the eye versus mouth area for the recognition of emotions varies across emotions (Beaudry et al., 2014). For example, happiness and disgust—which are associated with least negative age effects—are better identified by looking at the mouth region (Bassili, 1979; Calder et al., 2000), whereas the eyes region is crucial for the identification of anger, sadness, and fear (Bassili, 1979; Calder et al., 2000; Wegrzyn et al., 2017)—emotions older adults typically show difficulties with. However, most studies investigating the impact of gaze patterns on emotion recognition performance found no relation between gaze pattern and participants’ ability to correctly identify facial emotion expressions (for a review see, Grainger and Henry, 2020). Consistently, using a reverse correlation emotion categorization task (Bubbles paradigm) Smith et al. (2018) showed that younger (19–31 years) and older adults (69–80 years) use similar visual information to decode emotional expressions, especially from happy and fearful faces.

Difficulties with emotion recognition might also result from altered brain function in older adults. Age-related difficulties in the accuracy and speed of reading facial emotions might be caused by functional changes in frontal and temporal brain regions (see Ruffman et al., 2008). Possibly, a reduced amygdala activation in older compared to younger adults (Mather et al., 2004) and volume reductions in medial prefrontal areas that are critically involved in the recognition of negative emotions (e.g., fear and anger; Ruffman et al., 2008), such as the orbitofrontal cortex (OFC) and the anterior cingulate cortex (ACC; Mather, 2016), may explain why negative emotions are more affected by aging than positive emotions (cf. a review, Ruffman, 2020). However, studies that directly examined brain activation profiles have provided mixed results. Differences in brain activation between younger and older adults during emotion recognition tasks were found in the OFC (Williams et al., 2006; Ebner et al., 2012), in the ACC (Williams et al., 2006; Ebner et al., 2012; Ziaei et al., 2016) and generally in the mPFC (Williams et al., 2006; Ziaei et al., 2016). For example, fear stimuli evoked an increase in medial prefrontal activation with increasing age, whereas medial prefrontal activation to happy stimuli attenuated with age (Williams et al., 2006). Similarly, greater dorsomedial PFC (dmPFC) activity in response to angry (relative to happy) faces was more evident for older than for younger adults (Ebner et al., 2012). Hence, studies do not necessarily point to an increased or decreased activation of the brain with increasing age; indicating that differences in brain activation across the different studies may also be due to differences in external task demands (Ruffman, 2020).

Taken together, it is still not entirely clear which mechanisms might underlie the age-related decline in emotion recognition.

## Study 1

As outlined above, gaze patterns during face recognition tasks seem to change across the lifespan (e.g., Wong et al., 2005; Sullivan et al., 2007). Although some studies indicate that differences in gaze patterns may not fully explain poorer emotion recognition in older adulthood (for a review, Grainger and Henry, 2020), it is unclear how the relationship between gaze direction and recognition of specific emotions evolves across the adult lifespan and which role individual face parts play in the identification of emotions. Research suggests that changes in emotion recognition may already occur in middle adulthood (see Grainger and Henry, 2020), but so far, only few studies (Isaacowitz et al., 2007; Mill et al., 2009) have investigated changes in emotion recognition in this age group. Individuals seem to recognize emotions more easily from stimuli that present the whole face as compared to only the eyes or only the mouth (Calder et al., 2000; Calder and Jensen, 2005). Partially obscuring a face disrupts holistic face processing and disables the relational processing of specific facial features to other facial features (Maurer et al., 2002). Some studies have addressed gaze direction patterns in emotion recognition (for a review, Grainger and Henry, 2020), but to our knowledge no studies have compared younger and middle-aged adults’ ability to correctly identify emotions from separate facial areas.

The goal of our first study was therefore to systematically test the potential differential impact of separate facial areas versus the full face on basic emotion recognition in younger (20–24 years) and middle-aged adults (40–65 years). To this end, participants were presented with pictures of emotional expressions that either showed the full face, only the upper half of the face (eyes region: forehead to middle of nose) or the bottom half (mouth region: middle of nose to chin). Given first evidence of age-related decline in the ability to identify facial emotion expressions in middle adulthood (Ruffman et al., 2008; Grainger and Henry, 2020) and a steep decrease in the recognition of sadness and anger from the age of 31 onwards (Mill et al., 2009), we expected younger adults to outperform middle-aged adults across all emotions and specifically in the identification of sadness and anger. Across age groups, we predicted better performance when full faces were presented, as full faces provide more information about emotional states than single sections in which only some facial features (e.g., mouth region) are visible (Calder et al., 2000; Maurer et al., 2002; Calder and Jensen, 2005). Though, the relative importance of specific facial features for the identification of emotional expressions may vary depending on the specific emotion (Beaudry et al., 2014). Research suggests that older adults prefer to look at the mouth region, whereas younger adults focus on the eye region (e.g., Wong et al., 2005) which may make them more “trained” at recognizing emotions from this specific facial region. Therefore, we expected older adults to perform better in the “lower half” (i.e., mouth region) than in the “upper half” (i.e., eyes region) condition.

### Method

#### Participants

A total of 207 individuals filled in the online survey. Inclusion criteria were German as native language and for the younger adult group being aged between 20 and 25 and for the middle-aged adult group being aged between 40 and 65 years of age. Exclusion criteria were any history or the presence of neurological diseases, psychotic disorders, alcohol, or drug abuse. Twenty-four participants were excluded due to incomplete data, and 56 due to not being part of the age groups of interest. The final sample comprised 127 participants, 62 younger adults (age in years: *M* = 22.03, *SD* = 1.46) and 65 middle-aged adults (age in years: *M* = 53.49, *SD* = 6.22). Age groups differed significantly with regards to their age [*F*(1, 125) = 1505.19, *p* < 0.001, *η_p_*^2^ = 0.92] and gender [younger adults: 54 females, eight males; middle-aged adults: 43 females, 22 males; *χ*^2^(1) = 7.71, *p* = 0.007, *η_p_*^2^ = 0.25]. All participants gave written informed consent before taking part in the study. The study was approved by the local Ethics Committee. Participants were mainly recruited *via* the participant pool of the Department of Psychology of the Johannes-Gutenberg-University Mainz and through the wider social network of the experimenters. Participants were given course credit for completing the online survey.

#### Material

##### Emotion Recognition

**Figure 1:**
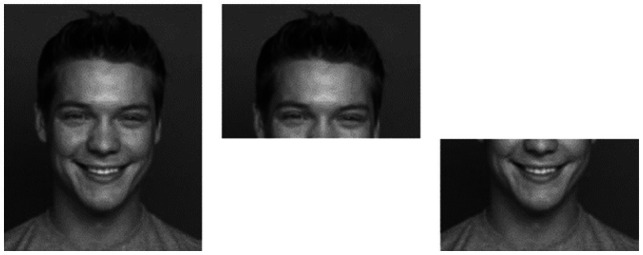
Shown are examples for images used for the emotion-recognition task. Six different emotions were presented either as picture of the full face or as upper/lower halves. Pictures here are “full happy face” (top left), “upper half” (middle), and “lower half” (bottom right). Modified and reproduced with permission from (Max Planck Institute for Human Development, Center for Lifespan Psychology, Berlin, Germany), available at (https://faces.mpdl.mpg.de/).

All emotion stimuli were obtained from the picture-set A of the FACES Database of the *Max-Planck-Institute for Human development* (Ebner et al., 2010), a validated set of pictures in terms of facial expressions displayed and age of the faces. Frontal photos of six Caucasians were used who belonged to three different age groups (i.e., young, middle-aged, and elderly; always one male and one female per age group). For each person, six different pictures were presented that displayed the emotional states angry, disgusted, fearful, happy, neutral, and sad. Stimuli of three different adult age groups were selected to minimize advantages or disadvantages of our younger and middle-aged participants when being presented with specific age groups (see Grundmann et al., 2021, for a similar approach). All pictures were presented in color. From each emotional expression once, the whole face was shown, once the lower and once the upper half of the face (upper half: forehead to middle of nose; lower half: middle of nose to chin; Figure 1). GIMP-GNU Image Manipulation Program was used to manipulate the pictures. In total, 108 pictures were presented in a randomized order. Each trial consisted of one picture. Underneath each picture the six emotion words (happy, anger, fear, neutral, sad, or disgust) were presented and participants were asked to choose *via* mouse click the emotion that was expressed in the picture. Each picture was presented until a response was made; there was no time limit. The raw dependent variable was the number of correctly identified emotions per emotion category and face section. Separate for each emotion category and face section *unbiased hit rates* (Hu) were calculated following Wagner (1993). Wagner’s formula aims to correct accuracy rates for chance (i.e., choosing the correct emotion label by chance) and answering habits (e.g., always selecting the response “happy” regardless of the presented emotion).

Given that previous research showed that specific emotions are often confused if only the eyes region is visible (Rinck et al., n.d.), we analyzed our data for confusion errors between angry and disgusted eyes as well as between sad and neutral eyes when participants were only presented with the upper face half. Similarly, previous research has shown that specific emotions are often confused when fixation on the mouth was enforced (e.g., Duran and Atkinson, 2021). Therefore, we analyzed our data for confusion errors between angry, disgusted, fearful, and sad mouths, respectively. For each of these pairs, we calculated the percentage of confusing one emotion as the other (e.g., percentage of reading angry eyes as disgusted eyes and the percentage of reading disgusted eyes as angry eyes).

#### Procedure

All data were collected anonymously *via* the online platform *SoSci Survey* (Leiner, 2019). After giving written informed consent, participants filled in questionnaires assessing sociodemographic information, and completed the emotion recognition task. The order of the emotional stimuli was randomized across participants. The entire procedure lasted about 45 min.

### Results

All statistical analyses were conducted with *IBM SPSS Statistics 27.0*.

**Figure 2:**
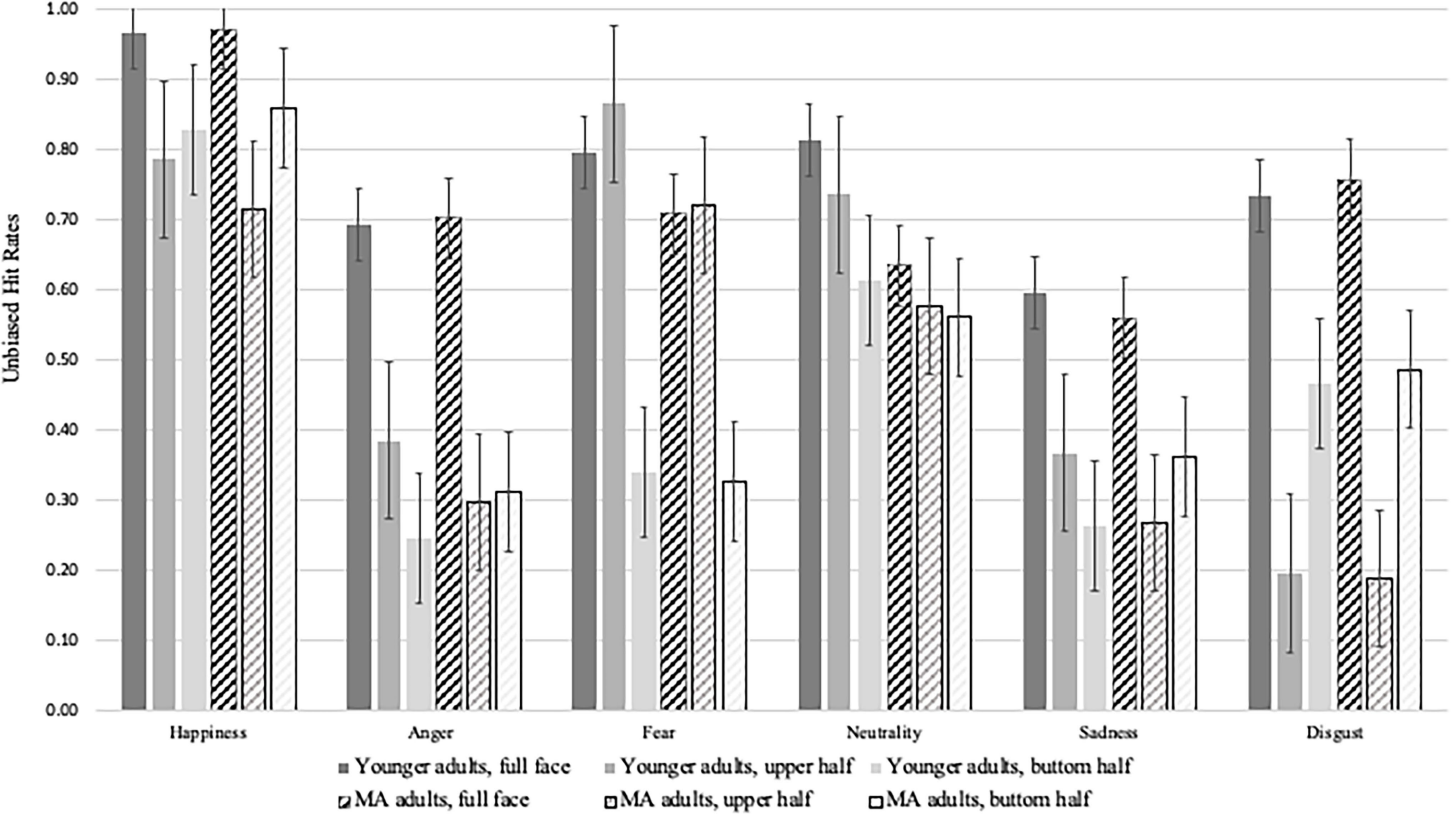
Emotion recognition performance per emotion, face section and age group.

To explore whether the effects of age group and the presented section of the face differed for the six presented emotions, we conducted a mixed 2 (between-subjects; age group: young/middle-aged) x 3 (within-subjects; face section: full face vs. lower half of the face vs. upper half of the face) x 6 (within-subjects; emotion category: happy, neutral, sad, angry, fearful, disgusted) ANOVA.^1^ If Mauchly’s Test of Sphericity indicated that the assumption of sphericity had been violated, Greenhouse–Geisser correction was applied. Means and standard errors can be taken from Figure 2.

There were significant main effects of age group [*F*(1,125) = 6.53, *p* = 0.012, *η*^2^*_p_* = 0.05], emotion category [*F*(4.23,625) = 349.42, *p* < 0.001, *η*^2^*_p_* = 0.74] and face section [*F*(2, 250) = 384.82, *p* < 0.001, *η*^2^*_p_* = 0.76]. These main effects were qualified by significant two-way interactions of age group by emotion [*F*(5,625) = 9.28, *p* < 0.001, *η*^2^*_p_* = 0.07], age group by face section [*F*(2, 250) = 15.97, *p* < 0.001, *η*^2^*_p_* = 0.11] and emotion by face section [*F*(7.97, 1,250) = 116.64, *p* < 0.001, *η*^2^*_p_* = 0.48] as well as a significant three-way interaction of age group, face section and emotion category [*F*(7.97, 1,250) = 1.91, *p* = 0.04, *η*^2^*_p_* = 0.02].

To better understand the three-way interaction, follow up analyses were conducted. Pairwise comparisons of age groups separately for each emotion and face section revealed the following: For happy expressions, younger adults only outperformed middle-aged adults when the upper half of the face was shown [*F*(1,125) = 4.96, *p* = 0.028, *η*^2^*_p_* = 0.04], but not when the lower half [*F*(1,125) = 2.52, *p* = 0.12, *η*^2^*_p_* = 0.02] or the full face was displayed (*F* < 1). Younger adults recognized more neutral expressions correctly from upper face halves and full faces than middle-aged adults [*F*(1,125) = 16.88, *p* < 0.001, *η*^2^*_p_* = 0.12 and *F*(1,125) = 21.35, *p* < 0.001, *η*^2^*_p_* = 0.15], while age groups did not differ when lower face halves were presented [*F*(1,125) = 3.78, *p* = 0.054, *η*^2^*_p_* = 0.03]. With regards to the detection of sad faces, middle-aged adults recognized more expressions correctly than younger adults when they were presented with lower face halves [*F*(1,125) = 9.44, *p* = 0.003, *η*^2^*_p_* = 0.07], while younger adults outperformed middle-aged adults when they were presented with upper face halves [*F*(1,125) = 9.79, *p* = 0.002, *η*^2^*_p_* = 0.07]; there were no age-related differences when the full face was displayed (*F* < 1.14). A similar pattern was observed for angry faces, also here middle-aged adults were better at recognizing the correct expressions from lower face halves [*F*(1,125) = 6.73, *p* = 0.011, *η*^2^*_p_* = 0.05], while younger adults outperformed older adults when presented with upper face halves [*F*(1,125) = 10.70, *p* = 0.001, *η*^2^*_p_* = 0.08]; there were no age-related differences when the full face was displayed (*F* < 1). For fearful faces, younger adults recognized more expressions correctly than middle-aged adults when they were presented with upper face halves [*F*(1,125) = 11.78, *p* < 0.001, *η*^2^*_p_* = 0.09] and full faces [*F*(1,125) = 5.57, *p* = 0.02, *η*^2^*_p_* = 0.04]; there were no age-related differences when lower face halves were displayed (*F* < 1). For the recognition of disgusted expressions, there were no age-related differences regardless of displayed face section (all *F*s < 1). After adjusting for multiple comparisons using Bonferroni correction (considering the intercorrelation of the variables, mean *r* = 0.156, see Sankoh et al., 1997, the corrected alpha level for each test is 0.004 to get an overall alpha level of 0.05) only the following effects were *no* longer significant between younger and middle-aged adults: neutral expressions when only upper face halves were shown, angry expressions when only lower face halves were shown and fearful expressions when full faces were presented.

Lastly, pairwise comparisons were conducted between the presented face sections, separately for each age group and each emotion. For younger adults, significant differences between the different face sections emerged for all emotions (all *p*s < 0.045), apart from happy expressions where no differences in recognition performance were found between lower and upper face halves. Except for fearful expressions, which were best recognized from upper face halves, all emotions were best recognized from full faces. Neutral, sad, and angry expressions were worst recognized from lower face halves, while happy and disgusted expressions were worst recognized from upper face halves. For middle-aged adults, significant differences between the different face sections emerged for all emotions (all *p*s < 0.011), except for neutral (no differences in recognition performance between lower and upper face halves and between upper halves and full faces), angry (no differences between lower and upper face halves) and fearful expressions (comparable detection rates when presented with upper and full faces). For all emotions, middle-aged adults showed best recognition performance when the full face was visible. Worst recognition rates for upper face halves were observed for happy, sad, angry, and disgusted expressions. Worst recognition rates for lower face halves were evident for neutral and fearful faces; here, performance did not differ from full face stimuli. After adjusting for multiple comparisons using Bonferroni correction (considering the intercorrelation of the variables, mean *r* = 0.156, see Sankoh et al., 1997, the corrected alpha level for each test is 0.004 to get an overall alpha level of 0.05) only the following effects were *no* longer significant: For younger adults, recognition rates did not differ anymore for neutral and fearful expressions when only upper face halves or full faces were presented. For middle-aged adults, recognition rates did not differ anymore for happy expressions when only upper face halves or full faces were presented.

#### Analyses of Confusion Errors

In a next step, we explored for specific emotions if there were age-related confusion errors when upper face halves were presented (see Table 1). *T* tests could not be computed for confusions of angry eyes as disgusted eyes, as only nine younger and six middle-aged adults committed this error. Younger adults confused disgusted eyes as angry eyes more often than middle-aged adults [*t*(115) = 2.37, *p* = 0.019, Cohen’s *d* = 0.44]. Overall, across age groups, participants rarely confused angry eyes as disgusted eyes, but rather frequently confused disgusted eyes as angry eyes. *T* tests indicated no age-related differences with regards to confusing sad eyes as neutral eyes [*t*(78) = 0.01, *p* = 0.995, Cohen’s *d* = 0.001], in contrast, middle-aged adults confused neutral eyes as sad eyes more often than younger adults [*t*(45.85) = −3.13, *p* = 0.003, Cohen’s *d* = −0.79]. Overall, across age groups, more confusion errors occurred for sad than for neutral eyes.

**Table 1:** Confusion errors.

	Younger adults *M* (*SD*)	Middle-aged adults *M* (*SD*)
Angry eyes as disgusted eyes	18.52% (5.56) *N* = 9	22.22% (8.61) *N* = 6
Disgusted eyes as angry eyes	53.67% (21.01) *N* = 59	44.25% (21.98) *N* = 58
Neutral eyes as sad eyes	19.79% (6.72) *N* = 16	28.65% (12.86) *N* = 32
Sad eyes as neutral eyes	27.93% (14.19) *N* = 37	27.91% (16.15) *N* = 43
Angry mouth as disgusted mouth	23.08% (10.62) *N* = 26	19.87% (8.19) *N* = 26
Disgusted mouth as angry mouth	16.67% (0.00) *N* = 7	21.60% (9.03) *N* = 27
Angry mouth as sad mouth	23.27% (9.99) *N* = 53	24.42% (9.86) *N* = 43
Sad mouth as angry mouth	19.89% (6.69) *N* = 31	20.29% (8.64) *N* = 23
Disgusted mouth as fearful mouth	17.54% (3.82) *N* = 19	19.30% (6.24) *N* = 19
Fearful mouth as disgusted mouth	28.51% (16.40) *N* = 38	26,75% (11.97) *N* = 38
Angry mouth as fearful mouth	16.67% (0.00) *N* = 2	16.67% (0.00) *N* = 5
Fearful mouth as angry mouth	18.51% (5.56) *N* = 9	18.75% (5.69) *N* = 16
Sad mouth as fearful mouth	20.00% (6.80) *N* = 25	21.05% (12.22) *N* = 19
Fearful mouth as sad mouth	22.22% (9.62) *N* = 3	19.44 (6.49) *N* = 12

In a further step, we explored for specific emotions if there were age-related confusion errors when lower face halves were presented (see Table 1). *T* tests could not be computed for confusions of disgusted mouths as angry mouths, as only seven younger adults, but 27 middle-aged adults committed this error. Likewise, *t* tests could not be computed for confusions of angry mouths as fearful mouths (two younger, five older adults) or fearful mouths as angry mouths (nine younger, 16 older adults) as well as fearful mouths as sad mouths (three younger, 12 older adults).

There were no significant age effects for any of the other confusion errors. Thus, middle-aged adults confused angry mouths as disgusted mouths [*t*(46.97) = 1.22, *p* = 0.23, Cohen’s *d* = −0.34], angry mouths as sad mouths [*t*(94) = −0.56, *p* = 0.58, Cohen’s *d* = 0.12] and sad mouths as angry mouths [*t*(52) = −0.19, *p* = 0.85, Cohen’s *d* = 0.05] as often as younger adults. Likewise, younger and older adults did not differ with regards to the confusion of disgusted mouths as fearful mouths [*t*(29.84) = −1.04, *p* = 0.31, Cohen’s *d* = 0.34], sad mouths as fearful mouths [*t*(42) = −0.36, *p* = 0.72, Cohen’s *d* = 0.11] and fearful mouths as disgusted mouths [*t*(74) = 0.53, *p* = 0.57, Cohen’s *d* = −0.12]. Overall, across age groups, most confusion errors occurred for angry mouths as sad mouths.

### Discussion

The aim of our first study was to investigate the impact of presenting different facial areas versus the full face on emotion recognition in younger (20–24 years) and middle-aged adults (40–65 years).

Our predictions were that younger adults would show significantly better emotion recognition than middle-aged adults, and that overall, participants would be better at identifying emotional expressions when full faces as compared to face halves were presented. As hypothesized, we did find a main effect of age group and a main effect of face section and both main effects were in the expected direction. However, given that these main effects were qualified by significant interactions, they could not be interpreted individually. We will therefore discuss the results considering the additional analyses following the significant three-way interaction of age group by face section by emotion category.

Importantly, younger adults did not outperform middle-aged adults across all emotions and presented face sections, but age-related effects varied depending on the presented emotion and face section. With regards to happy expressions, younger adults only showed more correct responses than middle-aged adults when the upper half of the face was presented, while younger and older adults did not differ in their performance when the lower half or the full face was displayed. These results fit nicely with Beaudry et al.’s (2014) study which indicated that the mouth area is particularly important in identifying happy expressions and with previous studies reporting no age-related deficits in the recognition of happy faces (e.g., Ruffman et al., 2008; Hayes et al., 2020). Younger adults correctly recognized more neutral and fearful expressions from upper face halves and full faces than middle-aged adults; age groups did not differ when lower face halves were presented. The spared performance of middle-aged adults when presented with fearful lower face halves may be due to their supposed preference to look at the mouth region (Wong et al., 2005), but also to a possibly strong distinctiveness of neutral and fearful expressions in the mouth area which may have enabled unimpaired performance (see also Grainger and Henry, 2020, for similar results of spared recognition of fearful and neutral faces by middle-aged adults). An interesting contrast was observed with regards to the detection of sad and angry faces, middle-aged adults recognized more sad and angry expressions correctly than younger adults when they were presented with lower face halves, while younger adults outperformed middle-aged adults when they were presented with upper face halves; there were no age-related differences when the full face was displayed. These findings are consistent with previous evidence indicating that useful visual information for sadness is present in both upper and lower face halves (Wegrzyn et al., 2017). The finding of spared identification of sad and angry faces when presented with full faces and even superior performance of middle-aged adults when being presented with lower face halves was in contrast to our expectations and previous evidence (see Mill et al., 2009; Hayes et al., 2020; but see Grainger and Henry, for similar results of spared recognition of sad expressions), as we had predicted age-related deficits in emotion recognition to be most pronounced for sadness and anger. However, at a second glance, the observed better performance of middle-aged adults when presented with sad or angry lower face halves and the observed better performance of younger adults when presented with upper face halves is in accords with previous studies indicating differential age-related looking patterns to the mouth (middle-aged/older adults) or to the eyes (younger adults; e.g., Wong et al., 2005; Sullivan et al., 2007). These differential age-related looking patterns may lead to more practice in detecting emotions from specific facial features which may underlie the observed differential age-related benefits in emotion recognition from specific face areas. However, this pattern of performance was evidently not found for all emotions; which may also result from the fact that emotions differ in terms of the informational value upper or lower facial features provide (Beaudry et al., 2014; Wegrzyn et al., 2017).

To investigate if some of the differences between the age groups might be the result of confusions between emotions when partial faces were presented,^2^ we tested for age-related differences in specific, most observed, confusion errors (Susskind et al., 2007; Langner et al., 2010) when being presented solely with the upper face halves (see Rinck et al., n.d., for a similar approach) or with the lower face halves (see Duran and Atkinson, 2021, for a similar approach). Middle-aged adults confused more often neutral eyes as sad eyes than younger adults, whereas the opposite error of confusing sad eyes as neutral eyes did not differ between age groups. Overall, across age groups, more confusion errors occurred for sad than for neutral eyes. Surprisingly, younger adults confused more often disgusted eyes as angry eyes than middle-aged adults, while there were no age effects with regards to the opposite error of confusing angry eyes as disgusted eyes. Overall, across age groups, participants rarely confused angry eyes as disgusted eyes, but rather frequently confused disgusted eyes as angry eyes. These findings are in line with Rinck and colleagues’ study who also reported clear response biases in the observed confusion errors. Their participants tended to misinterpreted disgust as anger, and sadness as neutral, whereas the opposite confusions occurred less often. These confusion errors are assumed to result from the fact that the expressions of anger and disgust as well as fear and neutrality share activation of facial muscles in the eyes area, but differ in the activation of facial muscles in the mouth area; with the latter not being visible in the upper face half condition (Smith et al., 2005; Wegrzyn et al., 2017). There were no age effects with regards to confusion errors when only the lower half of the face was presented. Overall, younger and middle-aged adults as frequently confused disgusted mouths as fearful mouths (or fearful as disgusted), sad mouths as fearful mouths, angry mouths as sad mouths (or sad as angry) and angry as disgusted mouths. Consistent with previous findings (e.g., Du and Martinez, 2011; Jack et al., 2014; Duran and Atkinson, 2021), presentation of the lower face half reduced the confusions of disgust as anger as compared to presentation of the upper face half. However, in absolute numbers more middle-aged adults confused disgusted mouths as angry mouths (younger adults *N* = 7, older adults *N* = 27), fearful mouths as angry (younger adults *N* = 9, older adults *N* = 16) or sad mouths than younger adults (younger adults *N* = 3, older adults *N* = 12) which argues against the assumption that middle-aged adults are more trained in recognizing emotions from lower face halves due to them focusing more on the mouth region (Wong et al., 2005).

In line with earlier research (e.g., Ruffman et al., 2008; Hayes et al., 2020), there were no age-related differences in the recognition of disgusted expressions—regardless of displayed face section. Overall, for both age groups and regardless of presented face section, recognition rates were best for happy and worst for disgusted expressions. In fact, performance was at ceiling for happy expressions and at floor for disgusted expressions which may have prevented the detection of age effects.

Following the theory of holistic facial processing (e.g., Calder et al., 2000; Maurer et al., 2002; Calder and Jensen, 2005) which states that facial emotion identification is disrupted when parts of the face are not visible for the observer, we had predicted that overall, participants would be better at recognizing emotions when the full face as compared to a “face section” (upper half or lower half) was presented. In line with previous studies indicating that the use of holistic identification strategies for emotion recognition does not decline with age (Konar et al., 2013; Meinhardt-Injac et al., 2017; Boutet and Meinhardt-Injac, 2019), middle-aged adults showed best recognition performance for all emotions when the full face was visible. Similarly, younger adults identified all emotions better from full faces, except for fearful expressions, which they recognized best from upper face halves. Interestingly and in line with assumptions of age-related differences in looking patterns, age groups differed with respect to the face *halves* from which they best recognized emotions. Middle-aged adults showed better recognition rates for happy, sad, angry, and disgusted expressions when lower as compared to upper face halves were presented; only neutral and fearful faces were better identified from upper than lower face halves. Younger adults only recognized happy and disgusted expressions better from lower than upper face halves, while neutral, sad, fearful, and angry expressions were better recognized from upper face halves as compared to lower face halves.

A potential limitation of Study 1 is the fact that gender distribution was not equal across age groups, but that there were more males in the sample of middle-aged adults. Given that males are found to have poorer emotion recognition performance than females (e.g., Wingenbach et al., 2018), this unequal distribution may have affected our results. Future studies should aim to test the same number of males and females in each age group. Importantly, when including gender as a factor into our analyses, we observed a main effect of gender, indicating that females outperformed males. However, there were no significant interactions of gender with any of the other factors, suggesting that gender did not affect the pattern of results.

Taken together, our results suggest that there is not a general decrease in emotion recognition across the lifespan (see Ruffman et al., 2008; Hayes et al., 2020), but that instead age-related differences depend on the specific emotion and presented face region. Furthermore, the results indicate that age-related differences in the correct identification of emotions can already be seen in middle-aged adults as compared to younger adults (e.g., Isaacowitz et al., 2007; Mill et al., 2009); though, age effects are contingent on face region.

## Study 2

Wearing face masks in everyday life is one of the consequences of the worldwide Covid-19 pandemic. Since the onset of the pandemic, the accompanying restrictions have had a major impact on our social lives. In many interpersonal interactions in public areas, we are now more dependent than ever on being able to recognize emotions from only the upper half of the face, as the other half is often covered by a mask. Our first study showed that emotion recognition is impaired in all age groups when only parts of the face are visible and information from other parts of the face are not available.

To date, several researchers around the world have studied the impact of mask wearing on the recognition of emotions across the entire lifespan. For example, Gori et al. (2021) investigated toddlers from 3- to 5-years, children from 6- to 8-years and adults from 18 to 30 years and found evidence for reduced emotion recognition when models were wearing a mask (versus not wearing a mask) in all age groups; though, particularly severe effects were seen in toddlers. In contrast, Ruba and Pollak (2020) reported no adverse effects of masks on children’s ability to recognize emotions when comparing participants’ ability to identify emotions from models wearing masks versus wearing sunglasses. Similarly, Calbi et al. (2021) showed spared emotion recognition in adults (mean age 36.2 years) when comparing pictures of models wearing face masks versus no masks. However, Marini et al. (2021) found face masks to negatively interfere with emotion recognition performance and trust attribution in adults (mean age 33 years). Consistently, Carbon (2020) reported reduced emotion recognition in healthy adults (range = 18–87 years) when models were wearing face masks; with the exception of fearful and neutral faces. Bani et al. (2021) also found worse emotion recognition from masked faces in a population of health care students (mean age 21.8 years; see Freud et al., 2020 for comparable results in adults, mean age 25.5 years) with more misattributions for happy and sad masked faces but no differences for fearful faces. The results of Parada-Fernández et al. (2022) showed that emotion recognition of all basic emotions (except for surprise) was more difficult for adults (range = 18–63 years, mean age 26.06 years), when the models wore masks. Grundmann et al. (2021) compared the identification of emotional facial expressions of younger (range = 19–31 years), middle-aged (range = 39–55 years) and older adults (range = 69–79 years) using a between-subject design and found the strongest negative impact of face masks on emotion recognition in older adults, suggesting that the consequences of wearing face masks in daily life can be particularly challenging for elderly people.

Taken together, most studies investigating the influence of face masks on facial emotion recognition found a significant decrease in emotion recognition for masked compared to unmasked faces (Carbon, 2020; Freud et al., 2020; Bani et al., 2021; Gori et al., 2021; Grundmann et al., 2021; Marini et al., 2021; Parada-Fernández et al., 2022), hence, wearing face masks restricts the recognition of emotions based on facial expressions. This decrease in emotion identification has also been shown by Study 1 and has been attributed to disruptions of holistic processing for faces with masks (Freud et al., 2020). Moreover, showing masked faces lowered participants’ confidence in their own assessment of the presented emotion (Carbon, 2020). However, interestingly, face masks seem to buffer against the detrimental effects of negative (vs. non-negative) emotion expressions on the perception of trustworthiness, likability, and closeness. Associating face masks with the dangers of the Covid-19 pandemic predicted a higher assessment of social closeness for masked, but not unmasked faces (Grundmann et al., 2021) and masked faces were rated as more trustworthy in general (Cartaud et al., 2020; Marini et al., 2021).

The goal of Study 2 was to investigate the effects of face masks on emotion recognition in younger and middle-aged to older adults in order to better understand possible difficulties in everyday communication due to the ongoing Covid-19 pandemic. In slight modification of Grundmann et al.’s study (2021), we applied a mixed design and presented younger (18–33 years) and middle-aged to older adults (51–83 years) with pictures of facial emotions that either showed the full face or faces wearing a medical face mask. Given evidence of age-related decline in the recognition of emotion expressions (see Ruffman et al., 2008), we expected the older age group to perform less well than younger adults. As emotions are more easily recognized in the context of the whole face compared with only the eyes or only the mouth (Calder et al., 2000; Calder and Jensen, 2005; also see our findings from Study 1) and first evidence of detrimental effects of face masks on emotion recognition (e.g., Carbon, 2020; Freud et al., 2020; Bani et al., 2021; Gori et al., 2021; Grundmann et al., 2021; Marini et al., 2021), we predicted better performance when unmasked faces were presented. Given previous evidence for older adults’ preference for looking at the mouth versus eyes region (and vice versa for younger adults; Wong et al., 2005), we expected middle-aged to older adults to be more disadvantaged in their ability to recognize emotions by masked faces than younger adults.

### Method

#### Participants

A total of 152 participants filled in the online survey. Inclusion criteria were German as native language and for the younger adult group being aged between 18 and 35 and for the middle-aged/older adult group being aged between 50 and 85 years of age. The older age group was a convenience sample and therefore covered a broader age range that comprised both, middle-aged and older adults. Exclusion criteria were any history or the presence of neurological diseases, psychotic disorders, alcohol, or drug abuse. Two participants were excluded due to incomplete data, and two due to not being part of the age groups of interest. Four participants were excluded because after having initially given informed consent before starting the online survey, they omitted the last question of the survey which asked them to agree again to having their data analyzed as part of the study. The final sample comprised 144 participants. Seventy-one participants were part of the younger adult group (age in years: *M* = 26.25, *SD* = 3.38, range 18–33 years; 53 females, 18 males) and 73 were part of the middle-aged/older adult group (age in years: *M* = 61.29, *SD* = 7.00, range 51–83; 55 females, 18 males). Age groups differed significantly with regards to their age [*F*(1, 142) = 1445.98; *p* < 0.001; *η*^2^*_p_* = 0.91], but not with respect to their education in years (younger adults *M* = 12.63, *SD* = 0.80, range 9–15; older adults *M* = 12.74, *SD* = 1.76, range 8–19; *F* < 1) or gender (for details see Table 2). All participants gave written informed consent before taking part in the study. The study was approved by the local Ethics Committee. Participants were mainly recruited *via* the participant pool of the Department of Psychology of the Johannes-Gutenberg-University Mainz and through the wider social network of the experimenters. Participants were not reimbursed for completing the online survey.

**Table 2:** Confusion errors.

	Younger adults *M* (*SD*)	Middle-aged/older adults *M* (*SD*)
Angry eyes as disgusted eyes	25.00% (0.00) *N* = 10	25.00% (0.00) *N* = 13
Disgusted eyes as angry eyes	50.00% (25.82) *N* = 61	50.83% (22.99) *N* = 60
Neutral eyes as sad eyes	25.00% (0.00) *N* = 2	26.19% (5.46) *N* = 21
Sad eyes as neutral eyes	27.27% (7.54) *N* = 11	25.00% (0.00) *N* = 13

#### Materials

##### Emotion Recognition

**Figure 3:**
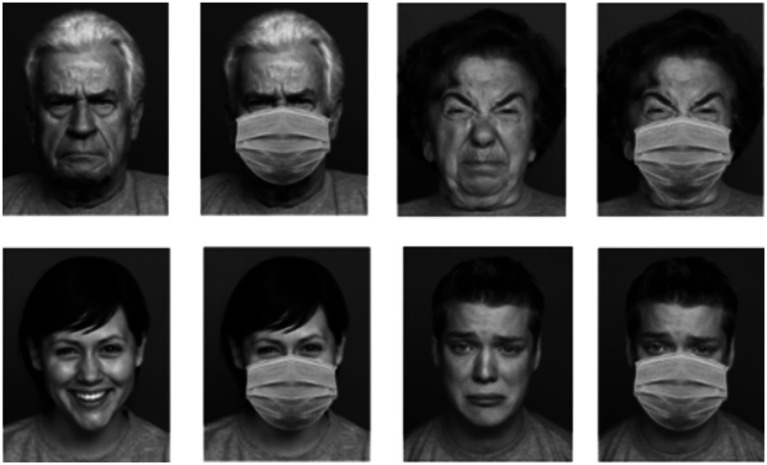
Shown are examples for images used for the emotion-recognition task. Six different emotions were presented with and without a face mask. Pictures here are “anger” (top left), “disgust” (top right), “happy” (bottom left), and “sad” (bottom right). Modified and reproduced with permission from (Max Planck Institute for Human Development, Center for Lifespan Psychology, Berlin, Germany), available at (https://faces.mpdl.mpg.de/).

All images of emotional expressions used in this study were younger and older, male or female adults from the picture-set B of the FACES Database of *Max-Planck-Institute for Human development* (Ebner et al., 2010); the picture-set had also been used by Grundmann et al. (2021). Microsoft PowerPoint was used to manipulate the pictures and thus to add the medical face mask. Pictures were presented in color. They showed one of the six emotions happy, anger, fear, neutral, sad or disgust (Figure 3). Each emotion was depicted once by a younger male and a younger female model as well as by an older male and an older female model. Each picture was presented once with and once without a face mask which resulted in 48 different pictures. Each trial consisted of one picture; pictures were presented in a randomized order. Underneath each picture the six emotion words (happy, anger, fear, neutral, sad or disgust) were presented and participants were asked to choose *via* mouse click the emotion that was expressed in the picture. Each picture was presented until a response was made; there was no time limit. The raw dependent variable was the number of correctly identified emotions per emotion category with a face mask and without a face mask. Separate for each emotion category and mask condition *unbiased hit rates* (Hu) were calculated following Wagner (1993).

To analyze for confusion errors between angry and disgusted as well as between sad and neutral eyes when being presented with masked faces (Rinck et al., n.d.), we calculated for each of these pairs the percentage of confusing one emotion as the other (e.g., percentage of reading angry eyes as disgusted eyes and the percentage of reading disgusted eyes as angry eyes).

#### Procedure

Data for this study were collected *via* an online questionnaire in *SoSci Survey* (Leiner, 2019) in two steps. After a short introduction, which mainly clarified the age groups of interest, potential participants were asked to enter their e-mail address. In a second step they received a personalized access link to the online survey. The survey started with an assessment of the sociodemographic data (i.e., age, gender, and education) of the participants. If a participant did not belong to one of the two target age groups, he or she was excluded at this point and could not continue with the questionnaire. After completing the sociodemographic questionnaire, emotion recognition was assessed.

### Results

All statistical analyses were conducted with *IBM SPSS Statistics 27.0*.

**Figure 4:**
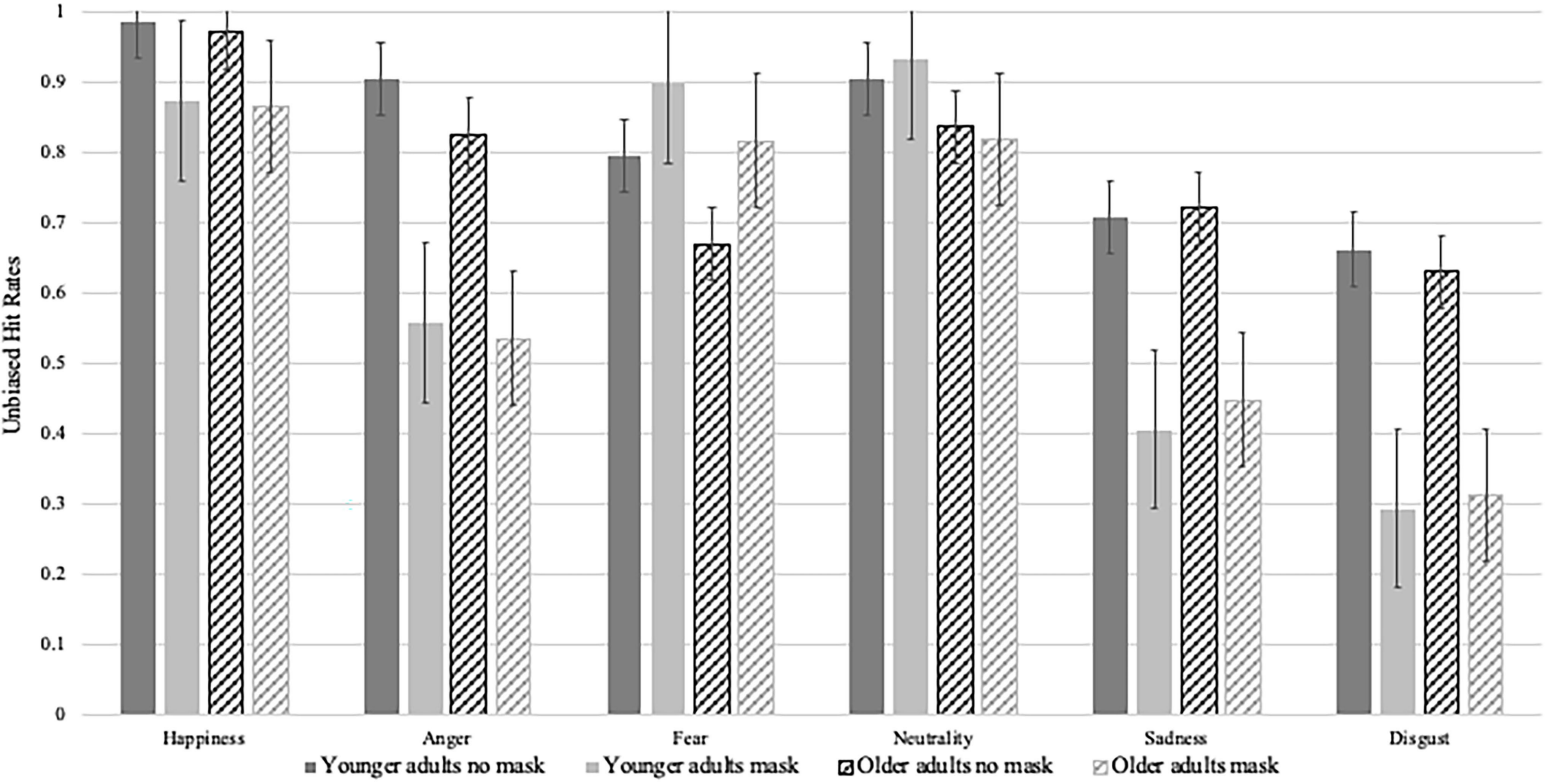
Emotion recognition performance per emotion, mask condition and age group.

To explore whether the unbiased hit rates of emotion recognition are influenced by age group, the presence or absence of face masks and emotion category, we conducted a mixed 2 (between subjects; age group: young/middle-aged to older) x 2 (within-subjects; face masks: present/absent) x 6 (within-subjects; emotions: happiness, anger, fear, neutral, sadness, disgust) ANOVA. Means and standard errors can be taken from Figure 4.

There was a significant main effect of the presence of masks [*F*(1,142) = 206.94, *p* < 0.001, *η_p_*^2^ = 0.59]; overall, participants showed better emotion recognition when stimuli were presented without a mask as compared to with a mask. There was no significant interaction of age group by the presence of masks (*F* < 1); thus, younger and middle-aged to older adults were not differentially impacted by the presence or absence of masks.

There were significant main effects of emotion category [*F*(3.92, 710) = 290.45, *p* < 0.001, *η_p_*^2^ = 0.67] and age group [*F*(1,142) = 9.60, *p* = 0.002, *η_p_*^2^ = 0.06]. These main effects were qualified by a significant interactions of emotion category by age group [*F*(3.92, 710) = 5.07, *p* < 0.001, *η_p_*^2^ = 0.03]. Further analyses of the interaction using simple effects only indicated significant age effects for the emotion categories anger [*F*(1,142) = 6.81, *p* = 0.01, *η_p_*^2^ = 0.05], fear [*F*(1,142) = 11.30, *p* < 0.001, *η_p_*^2^ = 0.07] and neutral [*F*(1,142) = 19.50, *p* < 0.001, *η_p_*^2^ = 0.12]. Younger adults outperformed middle-aged to older adults in the recognition of angry, fearful, and neutral expressions; there were no age-related differences with regards to the identification of happy, sad, and disgusted expressions. For younger adults, emotion recognition performance significantly differed between all emotion categories (all *p*s < =0.002); except for happy and neutral expressions (*p* = 0.489). Younger adults recognized happy and neutral expressions most frequently, followed by fearful, angry, sad, and disgusted expressions. For middle-aged to older adults, emotion recognition performance differed significantly between all emotion categories (all *p*s < =0.002). Middle-aged to older adults recognized happy expressions most frequently, followed by neutral, fearful, angry, sad, and disgusted expressions.

There was a significant interaction of the presence of masks and emotion categories [*F*(5, 710) = 113.73, *p* < 0.001, *η_p_*^2^ = 0.45]. Further analyses using simple effects revealed that participants’ ability to correctly identify emotions differed depending on the presence or absence of masks for happy, angry, fearful, sad, and disgusted expressions (all *p*s < 0.001); there was no significant difference for neutral stimuli (*F* < 1). Overall, participants recognized the emotions happiness, anger, sadness, and disgust less correctly when the model was wearing a face mask than when no face mask was present. In contrast, better emotion recognition was observed when a fearful expression was shown, and the model was wearing a mask.

Within the no face mask condition, recognition performance differed between all emotions (all *p*s < 0.001); except for neutral and angry stimuli as well as fearful and sad stimuli which were recognized equally well. Overall, participants recognized happy expressions most frequently, followed by neutral and angry expressions, fearful and sad expressions; disgusted expressions were least often identified correctly. Within the mask condition, recognition rates between happy and fearful, happy and neutral, fearful and neutral, fearful and sad did not differ, while there were significant differences for all other emotions (all *p*s < 0.001). Overall, participants recognized neutral expressions most often correctly, closely followed by happy and fearful expressions, which were followed by angry, sad, and disgusted expressions.

There was no significant three-way interaction of the presence of absence of face masks by emotion category by age group (*F* < 1).

#### Analyses of Confusion Errors

In a further step, we explored for specific emotions if there were age-related confusion errors when masked faces were presented (see Table 2). *T* tests indicated no age-related differences with regards to confusing disgusted eyes as angry eyes or sad eyes as neutral eyes (all *p*s > 0.29). *T* tests could not be computed for confusion errors of neutral eyes as sad eyes, as only two younger adults and 21 middle-aged to older adults committed this error. Furthermore, it was also not possible to conduct *t* tests for confusion errors of angry eyes as disgusted eyes as the SD was zero for both age groups. The most frequent confusion was that of disgusted eyes as angry eyes.

### Discussion

The aim of the second study was to investigate if wearing face masks differentially compromises younger (18–33 years) and middle-aged to older adults’ (51–83 years) facial emotion recognition performance.

In contrast to our expectations (e.g., Mill et al., 2009; Grainger et al., 2015), there was no general deficit in emotion recognition in middle-aged to older adults as compared to younger adults. In fact, significant age effects only emerged for the emotion categories anger, fear and neutral. Younger adults outperformed older adults in the recognition of angry, fearful, and neutral expressions; there were no age-related differences with regards to the identification of happy, sad, and disgusted expressions. The observed age-related deficits in the detection of angry and fearful expressions as well as the spared ability to detect happy and disgusted expressions are consistent with previous studies (e.g., Grainger and Henry, 2020). However, the lack of age effects with regards to the identification of sad stimuli was surprising and in contrast to earlier studies (Ebner and Johnson, 2009; Mill et al., 2009; Hayes et al., 2020).

Contrary to our expectation there was no significant interaction of age group by the presence vs. absence of face masks; hence, younger and middle-aged to older adults were comparably affected by face masks. This contrasts with the findings of Grundmann et al. (2021) who compared younger, middle-aged and older adults’ emotion recognition performance when being presented with masked or unmasked faces and even used the same stimuli dataset (i.e., FACES) as the present study. Grundmann and colleagues reported that older adults’ emotion recognition performance suffered most under masked faces. Importantly, the average age of the older adult’s group in the study of Grundmann et al. was about 10 years (*M* = 72.50) older than in our study (*M* = 61.29). It is therefore likely that the differences in study results can be explained by the lower average age of our participants.

Consistent with our predictions, participants recognized less emotions correctly when models were wearing a face mask versus when no face mask was worn, and the full face was visible. This is in line with previous research indicating that emotions are more easily recognized in the context of the whole face compared with only the eyes or only the mouth (so-called “holistic” processing; Calder et al., 2000; Calder and Jensen, 2005) and first evidence of detrimental effects of face masks on emotion recognition (e.g., Carbon, 2020; Freud et al., 2020; Bani et al., 2021; Gori et al., 2021; Grundmann et al., 2021; Marini et al., 2021). Furthermore, there was a significant interaction of the presence of masks and emotion categories. Overall, participants recognized the emotions happiness, anger, sadness, and disgust less often correctly when the model was wearing a face mask than when no face mask was present. In contrast, better emotion recognition was observed for fearful expressions when the model was wearing a mask, and detection rates did not differ for neutral expressions regardless of the presence of masks. These findings are partly inconsistent with previous studies (e.g., Carbon, 2020; Bani et al., 2021), which suggested that emotions like fear, anger and sadness are mainly expressed by the eyes region (Bassili, 1979; Calder et al., 2000; Wegrzyn et al., 2017) and should thus be more easily identified when models are wearing face masks and only the eyes region is visible avoiding distracting information. On the other hand, there is evidence that eyes do not always represent the most useful region for the detection of fearful and angry expressions when surprised and disgusted expressions are also present (see Smith and Merlusca, 2014). If disgusted expressions are present, angry expressions are frequently misinterpreted as disgusted if only the eyes area is visible. Possibly, the lower recognition rates of angry masked faces as compared to unmasked faces in our study are due to such confusion errors. Additional analyses indicate that about 50% of angry eyes were interpreted as disgusted eyes, while the opposite error hardly occurred (see Du and Martinez, 2011; Jack et al., 2014; Duran and Atkinson, 2021, for similar findings). Importantly, there were no age-related differences with regards to misjudging angry eyes as disgusted, whereas, more middle-aged to older adults confused disgusted eyes with angry eyes than younger adults. Given that only fearful, but no surprised faces were presented (with which fearful faces are typically confused; Smith and Merlusca, 2014), may have contributed to participants’ superior detection rates of fearful masked faces in the present study.

Taken together, present findings suggest that the information that can be read from the eyes is more limited than what was often assumed in the past (e.g., Baron-Cohen et al., 2001). Indeed, there is first evidence (Blais et al., 2012), that the mouth region may be more important for the correct identification of basic emotions and that the eyes region may be more relevant for the recognition of complex mental states (Baron-Cohen et al., 1997; see also Carbon, 2020, for a similar discussion on neutral expressions).

Regardless of whether masks were present or not, participants had most difficulties with the identification of disgusted faces, though, recognition rates were significantly lower when masked as compared to unmasked faces were presented. Within the no face mask condition, participants were best at recognizing happy expressions, whereas, in the mask condition, descriptively, neutral expressions were most often identified, closely (with no significant difference) followed by happy and fearful expressions.

Taken together, using a large sample (*N* = 144), present results confirm previous findings of reduced emotion recognition when the other person is wearing a face mask (e.g., Carbon, 2020; Freud et al., 2020; Bani et al., 2021; Grundmann et al., 2021; Marini et al., 2021). The restricted recognition of emotions could result in the misinterpretation of non-verbal, mimic communication with a wide range of consequences; however, further research is needed to investigate this. Based on the present findings it seems necessary to enhance non-verbal communication to prevent potential misinterpretation due to wearing face masks, for example, by means of verbal and additional non-verbal communication as well as context information.

## General Discussion

The ability to recognize emotions by interpreting another person’s non-verbal expressions is essential for effective communication (McArthur and Baron, 1983). The main aim of Study 1 and 2 was to explore possible age-related differences in emotion recognition when only parts of the face are visible. While in the past a restricted visibility of the face/facial mimic was hardly relevant for everyday life, due to the Covid-19 pandemic and the request to wear face masks indoors, we are now confronted with this situation daily.

In both studies, there was no general deficit of middle-aged to older adults in emotion recognition compared to younger adults (cf. meta-analyses, Ruffman et al., 2008; Hayes et al., 2020). Importantly, age-related differences in the identification of emotional expressions varied across emotions. Study 2 indicated overall superior performance of younger adults in the recognition of angry, fearful, and neutral expressions; there were no age-related differences with regards to the identification of happy, sad, and disgusted expressions. For Study 1, age-related differences in emotion recognition interacted with the presented facial area.

Across both studies emotion recognition was reduced when participants were only shown separate sections of faces versus full faces (i.e., Study 1: eyes or mouth region; Study 2: facial mask), with exception of fearful upper face halves for younger adults in Study 1. These results are consistent with the general assumption of holistic face processing (e.g., Calder et al., 2000; Maurer et al., 2002; Calder and Jensen, 2005) which seems to be age-independent (Konar et al., 2013; Meinhardt-Injac et al., 2017; Boutet and Meinhardt-Injac, 2019). Study 1 found that younger adults tended to read more emotions correctly from the upper half of the face, while middle-aged to older adults tended to identify emotions more often correctly from the lower half of the face, which may be concordant with earlier reports of differential preferred looking patterns of these age groups (e.g., Calder et al., 2000; Murphy and Isaacowitz, 2010; Grainger and Henry, 2020) and consequently more practice in the identification of emotions from these facial regions. However, Study 2 only observed better recognition of fearful and neutral masked faces (hence, analogous to upper face halves in Study 1) by younger adults as compared to middle-aged to older adults, while for all other (masked) emotional expressions performance was comparable for younger and older adults. This was somewhat surprising given that being presented with pictures of emotional expressions of models wearing face masks should pose similar demands on participants’ recognition skills as being presented with only the upper half of faces, as in both conditions similar parts of the face are visible while the rest is hidden under a mask or simply not presented (upper half condition). However, when we directly compare participants’ ability to correctly identify emotional expressions from upper face halves (Study 1) and masked faces (Study 2), we can see that both, younger and older adults’ performance was lower in Study 1 than Study 2—even though the older age group of Study 2 was on average 10 years older than the one of Study 1 and should thus be more affected by negative age effects. Possibly, due to the Covid-19 pandemic and the requirement to wear masks indoors, participants were more used to reading emotions from masked faces than from artificially cut face sections (Study 1).

Importantly, the results of Study 2 are also in contrast to Grundmann and colleagues’ findings who reported an interaction between age group and the presence or absence of masks. In Study 2, middle-aged to older adults’ emotion recognition performance was not more impaired by models wearing face masks than those of younger adults which supports previous evidence against a general effect of age-related gaze patterns on emotion recognition in younger and middle-aged to older adults.

Taken together, until to date, research has not been able to clarify which mechanisms underlie the age-related decline in emotion recognition. Similarly, neither Study 1 nor Study 2 fully support existing accounts of age-related differences in gaze direction patterns (e.g., Wong et al., 2005; Sullivan et al., 2007) or in valence-specific focus of attention (i.e., the positivity effect; Keightley et al., 2006; Orgeta and Phillips, 2008; Murphy et al., 2010; Murphy and Isaacowitz, 2010). Importantly, the finding that middle-aged to older adults more often confused neutral eyes with sad eyes than younger adults even speaks against the positivity bias. Further research using smaller age bands is needed to more accurately assess when age-related changes in emotion recognition emerge.

It is of note that the comparison of Study 1 and 2 is somewhat limited due to the two older age groups not comprising the same age range which may have differentially affected studies’ results and may restrict our conclusions. Furthermore, a potential limitation of the present studies is the use of static images as stimuli. There is evidence that more ecologically valid stimuli (e.g., dynamic stimuli) can improve emotion perception in younger and older adults (Ambadar et al., 2005; Grainger et al., 2015) and even lead to spared emotion recognition in older adults (Krendl and Ambady, 2010; Cortes et al., 2021). Therefore, the current studies may not accurately reflect individuals’ emotion recognition abilities in everyday life, where emotional expressions are naturally dynamic and embedded into a broader social interaction. Future studies should try to more closely mimic everyday life by using dynamic stimuli within a social context (see Hayes et al., 2020). Relatedly, future studies should use continuous emotion stimuli (rather than simply positive vs. negative valent stimuli) to allow for a more fine-grained approach in investigating age differences in emotion processing (Kunzmann et al., 2014).

In summary, the present studies point to a first decline in facial emotion recognition in middle adulthood. Both studies indicated worse emotion recognition (except in younger adults for fearful expressions), if only a separate area of the face (upper/lower half) is presented, and the face is not visible as a whole. The present results have practical as well as clinical implications. The rapid detection of emotional facial expressions is essential in social situations. Clinical studies on the health care of older patients have found that smiling and eye contact improves the quality of interactions in care settings (e.g., Ambady et al., 2002; de Vocht et al., 2015). Beneficial effects of emotional expressions may be reduced if faces of caretakers are covered by a face mask. On the other hand, facial expressions are only one part of human communication, body movement, gestures, or changes in the tone of voice also provide information about another person’s emotional state (e.g., Chen and Whitney, 2019).

## Data Availability Statement

The raw data supporting the conclusions of this article will be made available by the authors, without undue reservation.

## Ethics Statement

The studies involving human participants were reviewed and approved by the Ethics Committee of the Department of Psychology at the Johannes Gutenberg University Mainz. The patients/participants provided their written informed consent to participate in this study.

## Author Contributions

LE and PH carried out the data acquisition. MA, PH, and LF performed the data analysis. LF and MA drafted the manuscript. All authors contributed to the experimental design of the study, were involved in the interpretation of data, revised the manuscript critically, and approved the submitted version to be published.

## Conflict of Interest

The authors declare that the research was conducted in the absence of any commercial or financial relationships that could be construed as a potential conflict of interest.

## Publisher’s Note

All claims expressed in this article are solely those of the authors and do not necessarily represent those of their affiliated organizations, or those of the publisher, the editors and the reviewers. Any product that may be evaluated in this article, or claim that may be made by its manufacturer, is not guaranteed or endorsed by the publisher.

## Footnotes

^1^Given the unequal distribution of gender across the two age groups of Study 1, we repeated our analysis controlling for gender. Results indicated a significant main effect of gender (*p* = 0.01), with women showing better emotion recognition than men. However, gender did not significantly interact with any of our variables of interest, therefore, we decided to keep reporting our analyses as planned without including gender.

^2^We thank an anonymous reviewer for this suggestion.
