# Supplementary material for: The Effects of Separate Facial Areas on Emotion Recognition in Different Adult Age Groups: A Laboratory and a Naturalistic Study
Source: Front Psychol. 2022 Jun 30;13:859464. doi: 10.3389/fpsyg.2022.859464 (PMC9281501; doi:10.3389/fpsyg.2022.859464)
Supplement: Supplementary file 1 [file Data_Sheet_1.docx]

**Supplement**

**Study 1**

**Method**

*Material*

*Individual difference variables.* Participants were asked to indicate their anxiety and stress level due to the ongoing Covid-19 pandemic by answering three questions („Do you feel stressed because of the corona pandemic”, “Do you feel burdened because of the corona pandemic and “Are you afraid to get infected with the corona virus?”).

Furthermore, all participants completed the German version of Beck’s Depression Inventory II (BDI-II, Beck et al., 1996). The BDI-II is a widely used 21-item self-report inventory measuring the severity of depression in adolescents and adults.

**Results**

*Individual difference variables.* ANOVAs were conducted to test for age effects with respect to Covid-19-related stress and fears as well as depressive symptoms. Middle-aged adults experienced more stress than younger adults, whereas younger adults felt more burdened than middle-aged adults because of the Covid-19 pandemic. Moreover, younger participants’ depressive symptoms were significantly higher than those of middle-aged participants. In contrast, age groups did not differ regarding their fear to get infected with Covid-19 (see Table 1 for details).

Table 1. Individual difference variables

|  | Younger adults  *M* (*SD*) | Middle-aged adults  *M* (*SD*) | *F*(1, 125) | *P* | η_p_^2^ |
| --- | --- | --- | --- | --- | --- |
| Stress | 2.26 (.92) | 2.62 (.87) | 5.00 | .027 | .039 |
| Burden | 3.27 (.83) | 2.82 (.81) | 9.93 | .002 | .074 |
| Fear | 2.5 (.99) | 2.25 (.811) | 2.52 | .115 | .020 |
| Depression | 8.42 (6.29) | 4.75 (4.6) | 14.15 | .000 | .102 |

*Emotion recognition*. A 2x3x3 mixed analysis of variance (ANOVA) was carried out with the between-subjects factor age group (young/middle-aged), the within-subjects factor face section (full face/ lower half/upper half) and the within-subjects factor age of the model (younger/middle-aged/older). If Mauchly's Test of Sphericity indicated that the assumption of sphericity had been violated, the Greenhouse-Geisser correction was used. The dependent variable was the raw number of correctly identified emotions (see Table 2).

Table 2. Means and standard deviations of the raw number of correctly identified emotions

|  | Younger adults | Middle-aged adults |
| --- | --- | --- |
|  | *M* (*SD*) | *M* (*SD*) |
| Full face (max. 36) | 30.78 (2.31) | 29.69 (3.3) |
| Lower half (max. 36) | 22.95 (2.87) | 23.64 (2.65) |
| Upper half (max. 36) | 25.19 (3.33) | 22.38 (4.23) |
| Young models total (max. 36) | 28.18 (2.49) | 27.09 (2.92) |
| Middle-aged models total (max. 36) | 26.54 (2.57) | 25.4 (3.26) |
| Older models total (max. 36) | 23.27 (2.77) | 22.32 (3.17) |
| Younger models full face (max. 12) | 10.92 (1.08) | 10.52 (1.34) |
| Younger models lower half (max. 12) | 8.48 (1.07) | 8.38 (1.19) |
| Younger models upper half (max. 12) | 8.77 (1.46) | 8.18 (1.65) |
| Middle-aged models full face (max. 12) | 10.06 (.96) | 9.82 (1.35) |
| Middle-aged lower half (max. 12) | 7.97 (1.4) | 8.43 (1.32) |
| Middle-aged upper half (max. 12) | 8.52 (1.56) | 7.15 (1.72) |
| Older models full face (max. 12) | 7.90 (1.33) | 7.05 (1.7) |
| Older models lower half (max. 12) | 6.5 (1.34) | 6.83 (1.24) |
| Older models upper half (max. 12) | 8.87 (1.21) | 8.45 (1.40) |

There was a significant age effect with regards to emotion recognition (*F*(1, 125) = 6.15; *p* = .015; η_p_^2^ = 0.47). Overall, younger adults (*M*=78.94; *SD*= 6.57). recognized more emotional expressions correctly than middle-aged adults (*M*=75.73; *SD*= 7.98).

There was a significant main effect of face section (*F*(2, 212.37) = 162.98; *p* < .001; η_p_^2^ = .57); with more emotions being recognized when participants responded to full faces then when they responded to lower or upper halves. Bonferroni-adjusted post-hoc analyses revealed an overall effect (*p* < .01) for full faces compared to upper halves 1.05, 95%-CI [.88, 1.23]) and lower halves (1.61, 95%-CI [1.39, 1.83]) as well as an overall effect (*p* < .01) for upper halves compared to lower halves (.56, 95%-CI [.30, .82])

Furthermore, there was a significant main effect of model age (*F*(2, 250) = 203.83; *p* < .001; η_p_^2^ = .6). Bonferroni-adjusted post-hoc analyses revealed an overall effect (*p* < .01) for young compared to middle-aged (.55, 95%-CI [.36, .75]) and older faces (1.61, 95%-CI [1.42, 1.81]), but no difference in recognition between middle-aged and older faces.

There was also a significant interaction between age group and the presented face section (F(2, 250) = 16.91; *p* < .001; η_p_^2^ = .12). Further analysis of the interaction with tests of simple effects revealed that overall younger adults recognized emotions more often correctly than middle-aged adults when they responded to full faces (*F*(1,125) = 4.68 *p* = .032; η_p_^2^ = .04) and upper halves (*F*(1,125) = 17.15 *p* < .001; η_p_^2^ = .12). Separate analyses for each age group revealed that younger adults recognized emotions best when the full face was shown in comparison to only the upper (5.6, 95%-CI [4.56, 6.53]) or lower half (7.8, 95%-CI [6.95, 8.72]); *F*(1.78, 108.69) = 196.25; *p* < .001; η_p_^2^ = .76. Furthermore, they identified more emotions correctly from the upper than the lower half of the face (2.2, 95%-CI [1.08, 3.41]). Similarly, middle-aged adults recognized emotions best when full faces were shown compared to upper (7.3, 95%-CI [6.13, 8.48]) and lower halves (6.1, 95%-CI [4.95, 7.14]); *F*(2, 128) = 136.06; *p* < .001; η_p_^2^ = .68. In contrast to younger adults, older adults identified more emotions correctly from lower halves than from upper halves (1.3, 95%-CI [0.39, 2.48]).

There was a significant interaction between the age of the models and face section (*F*(4, 500) = 92.24; *p* < .001; η_p_^2^ = .43); with younger and middle aged models’ emotions being more often recognized when participants responded to full faces than half faces (*F*(2,365) = 211.15; *p* < .001). Older models’ emotional expressions were most frequently recognized when the upper half of the face was shown compared to full faces (1.21, 95%-CI [.95, 1.48]) and lower halves (2.1, 95%-CI [1.78, 2.41]), and full faces were more frequently recognized (.88, 95%-CI [.56, 1.21]) than lower halves (*F*(1.89; 525.17) = 140.76; *p* = .009; η_p_^2^ = .34).

At last, there was a significant three-way interaction between the age of the model presented, the participants’ age group and the presence or absence of a full face stimulus (*F*(4,500) = 5.09; *p* = .01; η_p_^2^ = .04).

There was no significant interaction between age group and age of the model (*F* < 1). Hence, younger and older adults recognized emotions of all age groups equally well.

*Report of all statistical values of the follow up analyses of the three-way interaction of age group, face section and emotion category*.

Table 3: Comparing younger and middle-aged adults’ emotion recognition performance per emotion and face section

| Emotions | Face region | *F* (1,125) | *p* | Partial eta squared |
| --- | --- | --- | --- | --- |
| Happy | Lower half | 2.522 | .115 | .020 |
|  | Upper half | 4.958 | .028 | .038 |
|  | Full face | .233 | .630 | .002 |
| Neutral | Lower half | 3.784 | .054 | .029 |
|  | Upper half | 16.882 | .000 | .119 |
|  | Full face | 21.346 | .000 | .146 |
| Sad | Lower half | 9.435 | .003 | .070 |
|  | Upper half | 9.791 | .002 | .073 |
|  | Full face | 1.138 | .288 | .009 |
| Angry | Lower half | 6.730 | .011 | .051 |
|  | Upper half | 10.703 | .001 | .079 |
|  | Full face | .107 | .744 | .001 |
| Fearful | Lower half | .120 | .729 | .001 |
|  | Upper half | 11.782 | .001 | .086 |
|  | Full face | 5.565 | .020 | .043 |
| Disgusted | Lower half | .304 | .582 | .002 |
|  | Upper half | .037 | .847 | .000 |
|  | Full face | .417 | .520 | .003 |

Table 4. Comparing emotions separately per face region and age group

|  |  |  |  |  |  |
| --- | --- | --- | --- | --- | --- |
| Face region | Emotions (I) | Emotions (J) | Mean Difference (I-J) | SE | p |
|  |  |  |  |  |  |
|  |  |  |  |  |  |
| **Younger adults** | |  |  |  |  |
| Lower half | Happy | Neutral | .213^*^ | .026 | .000 |
|  |  | Sad | .564^*^ | .027 | .000 |
|  |  | Angry | .581^*^ | .023 | .000 |
|  |  | Fearful | .489^*^ | .028 | .000 |
|  |  | Disgusted | .359^*^ | .029 | .000 |
|  | Neutral | Happy | -.213^*^ | .026 | .000 |
|  |  | Sad | .352^*^ | .027 | .000 |
|  |  | Angry | .368^*^ | .025 | .000 |
|  |  | Fearful | .276^*^ | .031 | .000 |
|  |  | Disgusted | .147^*^ | .032 | .000 |
|  | Sad | Happy | -.564^*^ | .027 | .000 |
|  |  | Neutral | -.352^*^ | .027 | .000 |
|  |  | Angry | .017 | .027 | .545 |
|  |  | Fearful | -.076^*^ | .032 | .019 |
|  |  | Disgusted | -.205^*^ | .028 | .000 |
|  | Angry | Happy | -.581^*^ | .023 | .000 |
|  |  | Neutral | -.368^*^ | .025 | .000 |
|  |  | Sad | -.017 | .027 | .545 |
|  |  | Fearful | -.092^*^ | .030 | .003 |
|  |  | Disgusted | -.222^*^ | .027 | .000 |
|  | Fearful | Happy | -.489^*^ | .028 | .000 |
|  |  | Neutral | -.276^*^ | .031 | .000 |
|  |  | Sad | .076^*^ | .032 | .019 |
|  |  | Angry | .092^*^ | .030 | .003 |
|  |  | Disgusted | -.129^*^ | .031 | .000 |
|  | Disgusted | Happy | -.359^*^ | .029 | .000 |
|  |  | Neutral | -.147^*^ | .032 | .000 |
|  |  | Sad | .205^*^ | .028 | .000 |
|  |  | Angry | .222^*^ | .027 | .000 |
|  |  | Fearful | .129^*^ | .031 | .000 |
| Upper half | Happy | Neutral | .051 | .030 | .092 |
|  |  | Sad | .419^*^ | .028 | .000 |
|  |  | Angry | .402^*^ | .026 | .000 |
|  |  | Fearful | -.080^*^ | .030 | .009 |
|  |  | Disgusted | .591^*^ | .029 | .000 |
|  | Neutral | Happy | -.051 | .030 | .092 |
|  |  | Sad | .368^*^ | .027 | .000 |
|  |  | Angry | .351^*^ | .030 | .000 |
|  |  | Fearful | -.130^*^ | .039 | .001 |
|  |  | Disgusted | .540^*^ | .035 | .000 |
|  | Sad | Happy | -.419^*^ | .028 | .000 |
|  |  | Neutral | -.368^*^ | .027 | .000 |
|  |  | Angry | -.018 | .025 | .492 |
|  |  | Fearful | -.499^*^ | .032 | .000 |
|  |  | Disgusted | .171^*^ | .032 | .000 |
|  | Angry | Happy | -.402^*^ | .026 | .000 |
|  |  | Neutral | -.351^*^ | .030 | .000 |
|  |  | Sad | .018 | .025 | .492 |
|  |  | Fearful | -.481^*^ | .032 | .000 |
|  |  | Disgusted | .189^*^ | .022 | .000 |
|  | Fearful | Happy | .080^*^ | .030 | .009 |
|  |  | Neutral | .130^*^ | .039 | .001 |
|  |  | Sad | .499^*^ | .032 | .000 |
|  |  | Angry | .481^*^ | .032 | .000 |
|  |  | Disgusted | .670^*^ | .035 | .000 |
|  | Disgusted | Happy | -.591^*^ | .029 | .000 |
|  |  | Neutral | -.540^*^ | .035 | .000 |
|  |  | Sad | -.171^*^ | .032 | .000 |
|  |  | Angry | -.189^*^ | .022 | .000 |
|  |  | Fearful | -.670^*^ | .035 | .000 |
| Full face | Happy | Neutral | .153^*^ | .026 | .000 |
|  |  | Sad | .369^*^ | .025 | .000 |
|  |  | Angry | .272^*^ | .021 | .000 |
|  |  | Fearful | .169^*^ | .028 | .000 |
|  |  | Disgusted | .231^*^ | .026 | .000 |
|  | Neutral | Happy | -.153^*^ | .026 | .000 |
|  |  | Sad | .217^*^ | .025 | .000 |
|  |  | Angry | .119^*^ | .030 | .000 |
|  |  | Fearful | .016 | .033 | .631 |
|  |  | Disgusted | .078^*^ | .032 | .017 |
|  | Sad | Happy | -.369^*^ | .025 | .000 |
|  |  | Neutral | -.217^*^ | .025 | .000 |
|  |  | Angry | -.098^*^ | .025 | .000 |
|  |  | Fearful | -.201^*^ | .027 | .000 |
|  |  | Disgusted | -.139^*^ | .026 | .000 |
|  | Angry | Happy | -.272^*^ | .021 | .000 |
|  |  | Neutral | -.119^*^ | .030 | .000 |
|  |  | Sad | .098^*^ | .025 | .000 |
|  |  | Fearful | -.103^*^ | .027 | .000 |
|  |  | Disgusted | -.041 | .023 | .083 |
|  | Fearful | Happy | -.169^*^ | .028 | .000 |
|  |  | Neutral | -.016 | .033 | .631 |
|  |  | Sad | .201^*^ | .027 | .000 |
|  |  | Angry | .103^*^ | .027 | .000 |
|  |  | Disgusted | .062^*^ | .027 | .023 |
|  | Disgusted | Happy | -.231^*^ | .026 | .000 |
|  |  | Neutral | -.078^*^ | .032 | .017 |
|  |  | Sad | .139^*^ | .026 | .000 |
|  |  | Angry | .041 | .023 | .083 |
|  |  | Fearful | -.062^*^ | .027 | .023 |
| **Middle-aged adults** | |  |  |  |  |
| Lower half | Happy | Neutral | .300^*^ | .025 | .000 |
|  |  | Sad | .499^*^ | .027 | .000 |
|  |  | Angry | .547^*^ | .022 | .000 |
|  |  | Fearful | .534^*^ | .027 | .000 |
|  |  | Disgusted | .374^*^ | .029 | .000 |
|  | Neutral | Happy | -.300^*^ | .025 | .000 |
|  |  | Sad | .199^*^ | .026 | .000 |
|  |  | Angry | .248^*^ | .024 | .000 |
|  |  | Fearful | .235^*^ | .030 | .000 |
|  |  | Disgusted | .074^*^ | .032 | .020 |
|  | Sad | Happy | -.499^*^ | .027 | .000 |
|  |  | Neutral | -.199^*^ | .026 | .000 |
|  |  | Angry | .049 | .027 | .071 |
|  |  | Fearful | .036 | .031 | .254 |
|  |  | Disgusted | -.125^*^ | .027 | .000 |
|  | Angry | Happy | -.547^*^ | .022 | .000 |
|  |  | Neutral | -.248^*^ | .024 | .000 |
|  |  | Sad | -.049 | .027 | .071 |
|  |  | Fearful | -.013 | .030 | .664 |
|  |  | Disgusted | -.173^*^ | .027 | .000 |
|  | Fearful | Happy | -.534^*^ | .027 | .000 |
|  |  | Neutral | -.235^*^ | .030 | .000 |
|  |  | Sad | -.036 | .031 | .254 |
|  |  | Angry | .013 | .030 | .664 |
|  |  | Disgusted | -.160^*^ | .030 | .000 |
|  | Disgusted | Happy | -.374^*^ | .029 | .000 |
|  |  | Neutral | -.074^*^ | .032 | .020 |
|  |  | Sad | .125^*^ | .027 | .000 |
|  |  | Angry | .173^*^ | .027 | .000 |
|  |  | Fearful | .160^*^ | .030 | .000 |
| Upper half | Happy | Neutral | .137^*^ | .029 | .000 |
|  |  | Sad | .446^*^ | .027 | .000 |
|  |  | Angry | .415^*^ | .026 | .000 |
|  |  | Fearful | -.007 | .029 | .802 |
|  |  | Disgusted | .524^*^ | .028 | .000 |
|  | Neutral | Happy | -.137^*^ | .029 | .000 |
|  |  | Sad | .309^*^ | .026 | .000 |
|  |  | Angry | .278^*^ | .029 | .000 |
|  |  | Fearful | -.145^*^ | .038 | .000 |
|  |  | Disgusted | .387^*^ | .034 | .000 |
|  | Sad | Happy | -.446^*^ | .027 | .000 |
|  |  | Neutral | -.309^*^ | .026 | .000 |
|  |  | Angry | -.031 | .025 | .210 |
|  |  | Fearful | -.454^*^ | .031 | .000 |
|  |  | Disgusted | .078^*^ | .031 | .014 |
|  | Angry | Happy | -.415^*^ | .026 | .000 |
|  |  | Neutral | -.278^*^ | .029 | .000 |
|  |  | Sad | .031 | .025 | .210 |
|  |  | Fearful | -.423^*^ | .031 | .000 |
|  |  | Disgusted | .109^*^ | .022 | .000 |
|  | Fearful | Happy | .007 | .029 | .802 |
|  |  | Neutral | .145^*^ | .038 | .000 |
|  |  | Sad | .454^*^ | .031 | .000 |
|  |  | Angry | .423^*^ | .031 | .000 |
|  |  | Disgusted | .532^*^ | .034 | .000 |
|  | Disgusted | Happy | -.524^*^ | .028 | .000 |
|  |  | Neutral | -.387^*^ | .034 | .000 |
|  |  | Sad | -.078^*^ | .031 | .014 |
|  |  | Angry | -.109^*^ | .022 | .000 |
|  |  | Fearful | -.532^*^ | .034 | .000 |
| Full face | Happy | Neutral | .337^*^ | .026 | .000 |
|  |  | Sad | .412^*^ | .025 | .000 |
|  |  | Angry | .269^*^ | .020 | .000 |
|  |  | Fearful | .263^*^ | .028 | .000 |
|  |  | Disgusted | .215^*^ | .026 | .000 |
|  | Neutral | Happy | -.337^*^ | .026 | .000 |
|  |  | Sad | .075^*^ | .024 | .002 |
|  |  | Angry | -.069^*^ | .029 | .019 |
|  |  | Fearful | -.074^*^ | .032 | .022 |
|  |  | Disgusted | -.122^*^ | .031 | .000 |
|  | Sad | Happy | -.412^*^ | .025 | .000 |
|  |  | Neutral | -.075^*^ | .024 | .002 |
|  |  | Angry | -.144^*^ | .024 | .000 |
|  |  | Fearful | -.149^*^ | .026 | .000 |
|  |  | Disgusted | -.197^*^ | .025 | .000 |
|  | Angry | Happy | -.269^*^ | .020 | .000 |
|  |  | Neutral | .069^*^ | .029 | .019 |
|  |  | Sad | .144^*^ | .024 | .000 |
|  |  | Fearful | -.005 | .026 | .834 |
|  |  | Disgusted | -.053^*^ | .023 | .022 |
|  | Fearful | Happy | -.263^*^ | .028 | .000 |
|  |  | Neutral | .074^*^ | .032 | .022 |
|  |  | Sad | .149^*^ | .026 | .000 |
|  |  | Angry | .005 | .026 | .834 |
|  |  | Disgusted | -.048 | .026 | .072 |
|  | Disgusted | Happy | -.215^*^ | .026 | .000 |
|  |  | Neutral | .122^*^ | .031 | .000 |
|  |  | Sad | .197^*^ | .025 | .000 |
|  |  | Angry | .053^*^ | .023 | .022 |
|  |  | Fearful | .048 | .026 | .072 |

Table 5. Comparing each emotion per face region and age group

| Emotions | Face region (I) | Face region (II) | Mean Difference (I-J) | SE | P |
| --- | --- | --- | --- | --- | --- |
|  |  |  |  |  |  |
|  |  |  |  |  |  |
| Younger adults | |  |  |  |  |
| Happy | Lower half | Upper half | .041 | .026 | .113 |
|  |  | Full face | -.139^*^ | .014 | .000 |
|  | Upper half | Lower half | -.041 | .026 | .113 |
|  |  | Full face | -.179^*^ | .024 | .000 |
|  | Full face | Lower half | .139^*^ | .014 | .000 |
|  |  | Upper half | .179^*^ | .024 | .000 |
| Neutral | Lower half | Upper half | -.121^*^ | .027 | .000 |
|  |  | Full face | -.198^*^ | .029 | .000 |
|  | Upper half | Lower half | .121^*^ | .027 | .000 |
|  |  | Full face | -.077^*^ | .034 | .024 |
|  | Full face | Lower half | .198^*^ | .029 | .000 |
|  |  | Upper half | .077^*^ | .034 | .024 |
| Sad | Lower half | Upper half | -.104^*^ | .031 | .001 |
|  |  | Full face | -.333^*^ | .033 | .000 |
|  | Upper half | Lower half | .104^*^ | .031 | .001 |
|  |  | Full face | -.229^*^ | .027 | .000 |
|  | Full face | Lower half | .333^*^ | .033 | .000 |
|  |  | Upper half | .229^*^ | .027 | .000 |
| Angry | Lower half | Upper half | -.138^*^ | .026 | .000 |
|  |  | Full face | -.448^*^ | .026 | .000 |
|  | Upper half | Lower half | .138^*^ | .026 | .000 |
|  |  | Full face | -.310^*^ | .022 | .000 |
|  | Full face | Lower half | .448^*^ | .026 | .000 |
|  |  | Upper half | .310^*^ | .022 | .000 |
| Fearful | Lower half | Upper half | -.527^*^ | .035 | .000 |
|  |  | Full face | -.458^*^ | .031 | .000 |
|  | Upper half | Lower half | .527^*^ | .035 | .000 |
|  |  | Full face | .069^*^ | .034 | .045 |
|  | Full face | Lower half | .458^*^ | .031 | .000 |
|  |  | Upper half | -.069^*^ | .034 | .045 |
| Disgusted | Lower half | Upper half | .272^*^ | .030 | .000 |
|  |  | Full face | -.267^*^ | .030 | .000 |
|  | Upper half | Lower half | -.272^*^ | .030 | .000 |
|  |  | Full face | -.540^*^ | .030 | .000 |
|  | Full face | Lower half | .267^*^ | .030 | .000 |
|  |  | Upper half | .540^*^ | .030 | .000 |
| Middle-aged adults | |  |  |  |  |
| Happy | Lower half | Upper half | .147^*^ | .025 | .000 |
|  |  | Full face | -.112^*^ | .014 | .000 |
|  | Upper half | Lower half | -.147^*^ | .025 | .000 |
|  |  | Full face | -.258^*^ | .023 | .000 |
|  | Full face | Lower half | .112^*^ | .014 | .000 |
|  |  | Upper half | .258^*^ | .023 | .000 |
| Neutral | Lower half | Upper half | -.015 | .027 | .564 |
|  |  | Full face | -.074^*^ | .029 | .011 |
|  | Upper half | Lower half | .015 | .027 | .564 |
|  |  | Full face | -.058 | .033 | .080 |
|  | Full face | Lower half | .074^*^ | .029 | .011 |
|  |  | Upper half | .058 | .033 | .080 |
| Sad | Lower half | Upper half | .095^*^ | .031 | .003 |
|  |  | Full face | -.198^*^ | .032 | .000 |
|  | Upper half | Lower half | -.095^*^ | .031 | .003 |
|  |  | Full face | -.293^*^ | .027 | .000 |
|  | Full face | Lower half | .198^*^ | .032 | .000 |
|  |  | Upper half | .293^*^ | .027 | .000 |
| Angry | Lower half | Upper half | .015 | .026 | .569 |
|  |  | Full face | -.390^*^ | .025 | .000 |
|  | Upper half | Lower half | -.015 | .026 | .569 |
|  |  | Full face | -.405^*^ | .021 | .000 |
|  | Full face | Lower half | .390^*^ | .025 | .000 |
|  |  | Upper half | .405^*^ | .021 | .000 |
| Fearful | Lower half | Upper half | -.395^*^ | .034 | .000 |
|  |  | Full face | -.383^*^ | .030 | .000 |
|  | Upper half | Lower half | .395^*^ | .034 | .000 |
|  |  | Full face | .012 | .033 | .714 |
|  | Full face | Lower half | .383^*^ | .030 | .000 |
|  |  | Upper half | -.012 | .033 | .714 |
| Disgusted | Lower half | Upper half | .297^*^ | .030 | .000 |
|  |  | Full face | -.270^*^ | .029 | .000 |
|  | Upper half | Lower half | -.297^*^ | .030 | .000 |
|  |  | Full face | -.568^*^ | .029 | .000 |
|  | Full face | Lower half | .270^*^ | .029 | .000 |
|  |  | Upper half | .568^*^ | .029 | .000 |

*Analyses of confusion errors*. T-tests comparing younger and middle-aged adults with regards to confusion errors.

Neutral eyes as sad eyes: t(45.85)=-3.132, p=.003, Cohen’s *d*= -0,789

Sad eyes as neutral eyes: t(78)=.006, p=.995, Cohen’s *d*=0,001

Disgusted eyes as angry eyes: t(115)= 2.370, p= .019, Cohen’s *d*= 0,438

Please note that it was not possible to run t tests on confusion errors of angry eyes as disgusted eyes as the number of participants committing this error was too little.

**Discussion**

Analyzes revealed a significant main effect of the model’s age. Participants of both age groups recognized emotions better if they were displayed by young models, closely followed by middle-aged faces. Emotions were most poorly recognized from older faces. However, no significant interaction between age group and model’s age was found; indicating no differential impact of model age on younger and middle-aged adults’ emotion recognition performance. These findings are consistent with previous studies reporting no general own-age bias (Borod et al., 2004; Ebner & Johnson, 2009; Malatesta, 1987; Riediger et al., 2011), but suggesting instead that all age groups find it harder to identify emotions from older faces compared to younger and middle-aged faces. Interestingly, the impact of models’ age on participants emotion recognition performance varied with the displayed face section (full faces versus separate face regions). Participants identified emotional expressions of younger and middle-aged models best when the full face was presented. In contrast, emotional expressions of older models were most often correctly recognized when only the upper half of the face was shown. These findings may be explained by age-related optical changes of the lower half of the face in older adults. Atrophy of the facial skeleton or the malposition of fatty muscles of the lower face represent typical age-related changes. Consequently, facial expressions in older people might be more difficult to recognize since they are wrinkled and have less control over their facial muscles as they get older (e.g., Albert et al., 2007). Therefore, participants may find it easier to recognize emotions from the upper halves of the face, as these are less affected by age-related visual changes.

**Study 2**

**Method**

*Individual difference variables.*  To assess participants’ attitude towards face masks, participants were required to indicate their agreement to eight statements on face masks (i. e. “I only wear a face mask because it is a legal requirement” or “I can protect myself and the people surrounding me if I wear a face mask”) using a 5-point-likert-scale ranging from “not at all” to “totally”. The dependent measure of attitude towards masks comprised a sum score of the eight face mask statements. Higher total scores indicate more positive attitudes and higher compliance towards wearing face masks.

To assess participants’ experienced anxiety and stress level with regards to the Covid-19 pandemic, they were asked to indicate their anxiety and stress level on two 5-point-likert-scale ranging from “not at all” to “extreme.

**Results**

*Individual difference variables*. ANOVAs were conducted to test for age effects with respect to Covid-19-related stress and fears as well as attitudes towards face masks. Overall, older adults were more afraid than younger adults to get infected with Covid-19. In contrast, age groups did not differ regarding the experienced stress due to the corona pandemic or their attitude towards face masks (see Table 6 for details).

Table 6. Individual difference variables

|  | Younger adults  *M* (*SD*) | Older adults  *M* (*SD*) | *F*(1, 142) | *p* | η_p_^2^ |
| --- | --- | --- | --- | --- | --- |
| Fear | 1.99 (.85) | 2.41 (.88) | 8.66 | .004 | .057 |
| Stress | 3.00 (.85) | 2.93 (1.06) | .18 | .676 | .001 |
| Attitude face masks | 28.1 (5.45) | 29.36 (5.40) | 1.94 | .166 | .013 |

*Emotion recognition*. A 2x2x2 mixed analysis of variance (ANOVA) was carried out with the between-subjects factor age group (young/older), the within-subjects factor presence or absence of a face mask on the presented picture and the within-subjects factor age of the model (young/older). The dependent variable was the raw number of correctly identified emotions (see Table 7).

Table 7. Means and standard deviations of the raw number of correctly identified emotions

|  | Younger adults | Older adults |
| --- | --- | --- |
|  | *M* (*SD*) | *M* (*SD*) |
| Total | 40.45 (2.44) | 39.08 (3.24) |
| No mask total (max. 24) | 21.52 (1.47) | 20.57 (1.56) |
| Mask total (max. 24) | 18.93 (1.57) | 18.33 (2.44) |
| Young models total (max. 24) | 21.49 (1.43) | 19.99 (1.83) |
| Older models total (max. 24) | 18.96 (1.69) | 19.1 (2.19) |
| Young models no mask (max. 12) | 11.03 (.96) | 10.04 (1.0) |
| Young models mask (max. 12) | 10.47 (1.09) | 9.95 (1.36) |
| Older models no mask (max. 12) | 10.49 (.98) | 10.71 (1.06) |
| Older models mask (max. 12) | 8.47 (1.09) | 8.38 (1.56) |

There was a significant age effect with regards to emotion recognition (*F*(1, 142) = 8.15; *p* = .005; η_p_^2^ = 0.54). Overall, younger adults recognized more emotional expressions correctly than older adults. There was a significant main effect of mask (*F*(1, 142) = 188.85; *p* = .000; η_p_^2^ = .57); with more emotions being recognized when the model was not wearing a face mask. Furthermore, there was a significant effect of model age; with participants showing more accurate performance when the model was young as compared to older (*F*(1, 142) = 87.69; *p* = .000; η_p_^2^ = .38). There was no significant interaction between age group and mask (*F* < 1). Hence, younger and older adults were comparably affected by models wearing masks. There was a significant interaction between the age of the model and the age group of the participant (*F*(1, 142) = 20.19; *p* = .000; η_p_^2^ = .12); with younger models’ emotions being more often recognized than older models’ by younger adults (*F*(1;142) = 30.16; *p* = .000; η_p_^2^ = .18). Older models’ emotional expressions were less frequently recognized than younger models’ emotional expressions; this was comparable for younger and older adults (*F*(1;142) = .18; *p* = .673; η_p_^2^ = .00).

There was a significant interaction between the age of the models presented and the presence versus absence of a face mask (*F*(1, 142) = 135.77; *p* = .000; η_p_^2^ = .49). Younger and older models’ emotions were recognized equally well if the model was not wearing a face mask. In contrast, if models were wearing face masks, younger models’ emotions were better recognized than older models’ emotions.

At last, there was a significant three-way interaction between the age of the model presented, the participants’ age group and the presence or absence of a face mask (*F*(1, 142) = 5.86; *p* = .017; η_p_^2^ = .04).

Table 8. Comparing younger and middle-aged to older adults per emotion

| Emotions | Mean Difference (younger – middle-aged/older adults) | SE | p |
| --- | --- | --- | --- |
|  |  |  |  |
| Happy | .012 | .015 | .418 |
| Angry | .053^*^ | .020 | .010 |
| Fearful | .104^*^ | .031 | .001 |
| Neutral | .092^*^ | .021 | .000 |
| Sad | -.001 | .024 | .963 |
| Disgusted | .006 | .026 | .807 |

Table 9. Comparing the different emotions per age group

| Emotions (I) | Emotions (J) | Mean Difference (I-J) | SE | p |
| --- | --- | --- | --- | --- |
|  |  |  |  |  |
| Younger adults |  |  |  |  |
| Happy | Angry | .196^*^ | .017 | .000 |
|  | Fearful | .083^*^ | .023 | .000 |
|  | Neutral | .010 | .015 | .489 |
|  | Sad | .346^*^ | .017 | .000 |
|  | Disgusted | .451^*^ | .020 | .000 |
| Angry | Happy | -.196^*^ | .017 | .000 |
|  | Fearful | -.113^*^ | .023 | .000 |
|  | Neutral | -.186^*^ | .019 | .000 |
|  | Sad | .150^*^ | .021 | .000 |
|  | Disgusted | .255^*^ | .016 | .000 |
| Fearful | Happy | -.083^*^ | .023 | .000 |
|  | Angry | .113^*^ | .023 | .000 |
|  | Neutral | -.073^*^ | .023 | .002 |
|  | Sad | .263^*^ | .025 | .000 |
|  | Disgusted | .368^*^ | .023 | .000 |
| Neutral | Happy | -.010 | .015 | .489 |
|  | Angry | .186^*^ | .019 | .000 |
|  | Fearful | .073^*^ | .023 | .002 |
|  | Sad | .335^*^ | .017 | .000 |
|  | Disgusted | .441^*^ | .023 | .000 |
| Sad | Happy | -.346^*^ | .017 | .000 |
|  | Angry | -.150^*^ | .021 | .000 |
|  | Fearful | -.263^*^ | .025 | .000 |
|  | Neutral | -.335^*^ | .017 | .000 |
|  | Disgusted | .105^*^ | .021 | .000 |
| Disgusted | Happy | -.451^*^ | .020 | .000 |
|  | Angry | -.255^*^ | .016 | .000 |
|  | Fearful | -.368^*^ | .023 | .000 |
|  | Neutral | -.441^*^ | .023 | .000 |
|  | Sad | -.105^*^ | .021 | .000 |
| Middle-aged to older adults | |  |  |  |
| Happy | Angry | .237^*^ | .017 | .000 |
|  | Fearful | .174^*^ | .022 | .000 |
|  | Neutral | .090^*^ | .014 | .000 |
|  | Sad | .332^*^ | .017 | .000 |
|  | Disgusted | .445^*^ | .019 | .000 |
| Angry | Happy | -.237^*^ | .017 | .000 |
|  | Fearful | -.062^*^ | .023 | .007 |
|  | Neutral | -.147^*^ | .019 | .000 |
|  | Sad | .096^*^ | .020 | .000 |
|  | Disgusted | .208^*^ | .016 | .000 |
| Fearful | Happy | -.174^*^ | .022 | .000 |
|  | Angry | .062^*^ | .023 | .007 |
|  | Neutral | -.085^*^ | .023 | .000 |
|  | Sad | .158^*^ | .024 | .000 |
|  | Disgusted | .271^*^ | .022 | .000 |
| Neutral | Happy | -.090^*^ | .014 | .000 |
|  | Angry | .147^*^ | .019 | .000 |
|  | Fearful | .085^*^ | .023 | .000 |
|  | Sad | .243^*^ | .017 | .000 |
|  | Disgusted | .355^*^ | .023 | .000 |
| Sad | Happy | -.332^*^ | .017 | .000 |
|  | Angry | -.096^*^ | .020 | .000 |
|  | Fearful | -.158^*^ | .024 | .000 |
|  | Neutral | -.243^*^ | .017 | .000 |
|  | Disgusted | .113^*^ | .021 | .000 |
| Disgusted | Happy | -.445^*^ | .019 | .000 |
|  | Angry | -.208^*^ | .016 | .000 |
|  | Fearful | -.271^*^ | .022 | .000 |
|  | Neutral | -.355^*^ | .023 | .000 |
|  | Sad | -.113^*^ | .021 | .000 |

Table 10. Comparing emotion recognition performance for masked versus non-masked expressions per emotion

| Emotions | Mean Difference (mask - no-mask) | SE | p |
| --- | --- | --- | --- |
|  |  |  |  |
| Happy | .110^*^ | .015 | .000 |
| Angry | .321^*^ | .018 | .000 |
| Fearful | -.125^*^ | .021 | .000 |
| Neutral | -.004 | .018 | .821 |
| Sad | .260^*^ | .019 | .000 |
| Disgusted | .343^*^ | .024 | .000 |

Table 11. Comparing emotions for masked versus non-masked faces

| Emotions (I) | Emotions (J) | Mean Difference (I-J) | SE | p |
| --- | --- | --- | --- | --- |
|  |  |  |  |  |
| With face masks |  |  |  |  |
| Happy | Angry | .111^*^ | .012 | .000 |
|  | Fearful | .246^*^ | .021 | .000 |
|  | Neutral | .107^*^ | .013 | .000 |
|  | Sad | .264^*^ | .015 | .000 |
|  | Disgusted | .331^*^ | .016 | .000 |
| Angry | Happy | -.111^*^ | .012 | .000 |
|  | Fearful | .135^*^ | .023 | .000 |
|  | Neutral | -.004 | .017 | .808 |
|  | Sad | .153^*^ | .019 | .000 |
|  | Disgusted | .221^*^ | .015 | .000 |
| Fearful | Happy | -.246^*^ | .021 | .000 |
|  | Angry | -.135^*^ | .023 | .000 |
|  | Neutral | -.139^*^ | .024 | .000 |
|  | Sad | .018 | .025 | .475 |
|  | Disgusted | .086^*^ | .019 | .000 |
| Neutral | Happy | -.107^*^ | .013 | .000 |
|  | Angry | .004 | .017 | .808 |
|  | Fearful | .139^*^ | .024 | .000 |
|  | Sad | .157^*^ | .016 | .000 |
|  | Disgusted | .225^*^ | .020 | .000 |
| Sad | Happy | -.264^*^ | .015 | .000 |
|  | Angry | -.153^*^ | .019 | .000 |
|  | Fearful | -.018 | .025 | .475 |
|  | Neutral | -.157^*^ | .016 | .000 |
|  | Disgusted | .068^*^ | .019 | .000 |
| Disgusted | Happy | -.331^*^ | .016 | .000 |
|  | Angry | -.221^*^ | .015 | .000 |
|  | Fearful | -.086^*^ | .019 | .000 |
|  | Neutral | -.225^*^ | .020 | .000 |
|  | Sad | -.068^*^ | .019 | .000 |
| Without face masks | |  |  |  |
| Happy | Angry | .322^*^ | .019 | .000 |
|  | Fearful | .011 | .020 | .570 |
|  | Neutral | -.007 | .016 | .660 |
|  | Sad | .414^*^ | .017 | .000 |
|  | Disgusted | .564^*^ | .023 | .000 |
| Angry | Happy | -.322^*^ | .019 | .000 |
|  | Fearful | -.310^*^ | .019 | .000 |
|  | Neutral | -.329^*^ | .019 | .000 |
|  | Sad | .092^*^ | .018 | .000 |
|  | Disgusted | .242^*^ | .018 | .000 |
| Fearful | Happy | -.011 | .020 | .570 |
|  | Angry | .310^*^ | .019 | .000 |
|  | Neutral | -.018 | .018 | .307 |
|  | Sad | .403^*^ | .018 | .000 |
|  | Disgusted | .553^*^ | .024 | .000 |
| Neutral | Happy | .007 | .016 | .660 |
|  | Angry | .329^*^ | .019 | .000 |
|  | Fearful | .018 | .018 | .307 |
|  | Sad | .421^*^ | .015 | .000 |
|  | Disgusted | .571^*^ | .023 | .000 |
| Sad | Happy | -.414^*^ | .017 | .000 |
|  | Angry | -.092^*^ | .018 | .000 |
|  | Fearful | -.403^*^ | .018 | .000 |
|  | Neutral | -.421^*^ | .015 | .000 |
|  | Disgusted | .150^*^ | .021 | .000 |
| Disgusted | Happy | -.564^*^ | .023 | .000 |
|  | Angry | -.242^*^ | .018 | .000 |
|  | Fearful | -.553^*^ | .024 | .000 |
|  | Neutral | -.571^*^ | .023 | .000 |
|  | Sad | -.150^*^ | .021 | .000 |

*Analyses of confusion errors*. T-tests comparing younger and middle-aged to older adults with regards to confusion errors.

Disgusted eyes as angry eyes: *t*(119)=.187, *p*=.852, Cohen’s *d*= -0,034

Sad eyes as neutral eyes *t*(22)=1.092, *p*=.287, Cohen’s *d*= 0,447

Please note that it was not possible to test for age effects between younger and middle-aged to older adults with regards to confusion errors, as only 2 younger and 21 middle-aged to older adults committed this error. It was also not possible to conduct the t test for confusion errors of angry eyes as disgusted eyes as the standard deviation of both age groups was zero.

**Discussion**

Consistent with Study 1, both, younger and older adults recognized younger models’ emotions better than older models’ emotions. Furthermore, there was a significant interaction between the age of the models presented and the age group of the participant. Younger adults performed significantly better in the emotion recognition task when emotions were displayed by younger models, while age groups did not differ in identifying emotions in older models. Younger adults were more able to recognize emotions displayed by younger models than older adults, even when the model was wearing a face mask. Hence, similarly to Study 1, these findings suggest no general own-age bias (see Borod et al., 2004; Ebner & Johnson, 2009; Malatesta, 1987; Riediger et al., 2011, for similar findings), but only an own-age bias for younger adults. The lack of an own-age bias for older adults may be due to the cumulative life experience of older adults’ in emotion recognition of faces of all age groups (Rhodes & Anastasi, 2012) or simply to the omnipresence of younger faces in the media.
